# Multi‐Stability of the Extensible Origami Structures

**DOI:** 10.1002/advs.202303454

**Published:** 2023-08-08

**Authors:** Kaili Xi, Sibo Chai, Jiayao Ma, Yan Chen

**Affiliations:** ^1^ Key Laboratory of Mechanism Theory and Equipment Design of Ministry of Education School of Mechanical Engineering Tianjin University 135 Yaguan Road Tianjin 300350 China

**Keywords:** kinematics, multi‐stability, non‐rigid foldability, programmability, wrapping origami

## Abstract

Multi‐stable structures and metamaterials with more than two stable states are widely applied in diversified engineering applications. Non‐rigid foldable origami patterns have provided an effective way of designing multi‐stable structures. But most of them have only two stable states and therefore require a combination of many units to achieve multi‐stability. Here, a series of extensible origami structures are proposed with generic multi‐stability based on non‐rigid wrapping origami. Through a kinematic analysis and experiments, it is demonstrate that a sequential folding among different layers of the structures is created to generate a continuous rigid origami range and several discrete rigid origami states, which consequently leads to the multi‐stability of the extensible origami structures. Moreover, the effects of design parameters on the mechanical properties of the structures are investigated by numerical simulation, enabling properties programmability upon specific needs. This study thus paves a new pathway for the development of novel multi‐stable origami structures.

## Graphical abstract

A series of extensible origami structures are proposed with generic multi‐stability based on non‐rigid wrapping origami. The sequential folding among different layers of the structures is proven to be crucial for their multi‐stability. Moreover, the effects of design parameters on the mechanical properties of the structures are investigated by numerical simulation, enabling properties programmability upon specific needs.

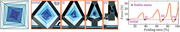

## 1 Introduction

Bistable/multi‐stable structures and metamaterials can rapidly switch among two or more stable states with distinct geometrical configurations and mechanical properties, and absorb a considerable amount of energy when overcoming the energy barrier between two states. Taking advantage of this unique feature, they have been widely utilized in numerous engineering applications, such as reconfigurable robotics,^[^ ^1^, ^2^ ^]^ actuators,^[^ ^3^, ^4^ ^]^ MEMS devices,^[^ ^5^, ^6^ ^]^ frequency programmable antenna,^[^ ^7^ ^]^ mechanical logic,^[^ ^8^, ^9^ ^]^ mechanical memory storage,^[^ ^10^, ^11^ ^]^ mechanical signals propagation,^[^ ^12^, ^13^, ^14^ ^]^ rapid deployment,^[^ ^15^, ^16^, ^17^, ^18^ ^]^ energy harvesting, and energy absorption.^[^ ^19^, ^20^, ^21^, ^22^ ^]^

Origami is an ancient art of folding flat sheets of paper to form complex two‐dimensional (2D) or 3D structures without cutting or bonding. Though traditional origami articles mainly originate from art, nowadays various design approaches, including periodic^[^ ^23^ ^]^ and non‐periodic^[^ ^24^ ^]^ tessellation, assignment of mountain‐valley crease,^[^ ^25^ ^]^ think‐panel transformation,^[^ ^26^ ^]^ biomimicry,^[^ ^27^ ^]^ have been proposed to develop novel origami structures with remarkable mechanical properties for applications in engineering fields from aerospace,^[^ ^28^, ^29^ ^]^ robotics,^[^ ^30^, ^31^ ^]^ metamaterials.^[^ ^32^, ^33^ ^]^ Alongside, series of analytical and numerical tools, including kinematics,^[^ ^34^ ^]^ computational geometry,^[^ ^35^, ^36^ ^]^ and crystallography,^[^ ^37^ ^]^ have also been utilized for the design and analysis of origami structures. Depending on the folding mechanics, origami can be categorized into rigid origami where facets are only allowed to revolve around creases without any deformation during the folding process,^[^ ^38^, ^39^ ^]^ and non‐rigid origami that requires crease rotation and facet distortion. From the viewpoint of energy, if we ignore the stiffness of the creases, a rigid origami folding process is always energy free. On the contrary, a non‐rigid origami pattern generally has two zero‐energy states, i.e., the initial states that are usually fully flattened, and the folded states that could be another flattened state^[^ ^40^, ^41^ ^]^ or a 3D one,^[^ ^42^, ^43^ ^]^ and an energy barrier needs to be overcome to transform between the two states. This unique feature makes them naturally suitable for the creation of bistable structures, typical examples including the non‐rigid type 1 and type 2 square‐twist,^[^ ^44^, ^45^ ^]^ Kresling,^[^ ^46^, ^47^ ^]^ wrapping origami,^[^ ^42^, ^43^ ^]^ Flasher origami.^[^ ^48^, ^49^ ^]^ However, there are usually only two stable states in a single structure. Consequently, to achieve multi‐stability, a common approach is to stack many bistable units to form large 1D,^[^ ^14^, ^50^, ^51^, ^52^, ^53^ ^]^ 2D,^[^ ^54^ ^]^ or 3D^[^ ^55^ ^]^ structures or metamaterials. To overcome this limitation, efforts have been made to develop generic multi‐stable structures out of non‐rigid origami. For instance, taking advantage of the bifurcation of the square‐twist pattern at the initial flattened state, a tri‐stable origami structure^[^ ^56^ ^]^ with two folded stable states and one unfolded stable state was created. Following a similar principle, Chen et al.^[^ ^57^ ^]^ found that hexagonal origami hypar, the name of which is derived from the combination of “hyperbolic” and “parabolic”,^[^ ^58^ ^]^ has three bifurcation paths at the fully flattened state and therefore could achieve six stable states. Nevertheless, such designs require sophisticated deformation control at the bifurcation point to reach the target stable states, which may pose restraints to their applications in certain scenarios.

Given this, here in this paper, we propose a series of generic multi‐stable origami structures based on non‐rigid foldable wrapping origami.^[^ ^42^, ^59^, ^60^ ^]^ By purposely modifying the crease pattern and extending it radially, we manage to tailor the folding sequence of different circumferential layers, thereby creating multiple stable states in a single structure that can be reached by a simple uniaxial loading. This work thus provides a useful design pathway for the development of novel multi‐stable origami structures to fulfill advanced engineering applications.

## 2 Results

### 2.1 Geometric Design of the Extensible Wrapping Origami

**Figure 1:**
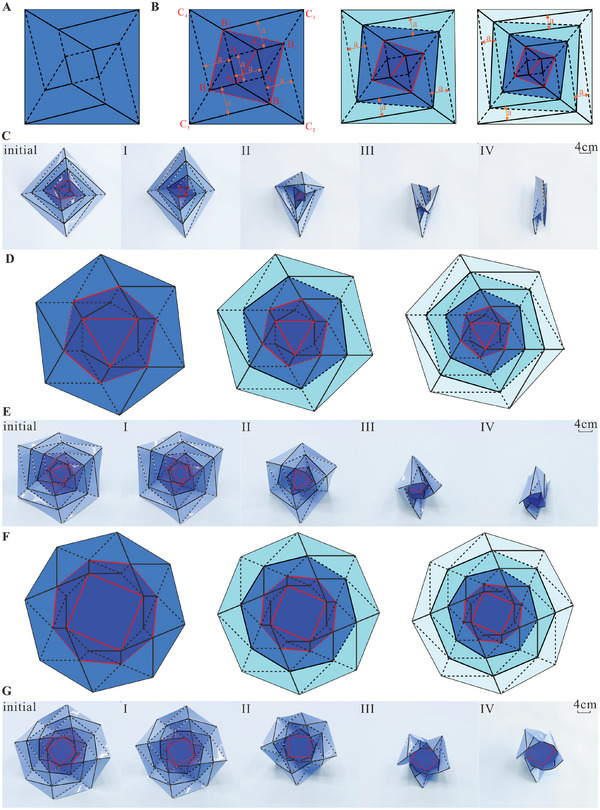
The crease patterns and physical specimens of the square, hexagonal, and octagonal extensible wrapping origami. A) The original crease pattern of the wrapping origami with the square hub. The solid and dashed lines represent the mountain and valley lines, respectively. B) The crease patterns of the two‐layer, three‐layer, and four‐layer square wrapping origami structures. C) The folding process of the four‐layer square wrapping origami physical specimen. The specimen with *a* = 30 mm and panel thickness *t* = 0.5 mm was manufactured from polyethylene glycol terephthalate (PET) and reinforced at panels by 304 stainless steel sheets. Due to the elastic spring back of the creases, the initial state of the specimen is not the fully deployed state. The initial state and states II to IV are the stable states, and folding encounters noticeable resistance starting at state I. D) The crease pattern of the two‐layer, three‐layer, and four‐layer hexagonal wrapping origami structures. E) The folding process of the four‐layer hexagonal physical specimen. The specimen was made from PET with *a* = 30 mm and *t* = 0.4 mm. F) The crease pattern of the two‐layer, three‐layer, and four‐layer octagonal wrapping origami structures. G) The folding process of the four‐layer octagonal physical specimen. The specimen was made from PET with *a* = 30 mm and *t* = 0.4 mm.

The geometry for the extensible wrapping origami is illustrated in **Figure** **1**. First, starting from the original wrapping origami pattern shown in Figure 1A, five new creases, one in the central square hub and four in the peripheral trapezoid facets, all of which are marked in red in the left subfigure of Figure 1B, are added to construct the crease pattern of the square wrapping origami. Then the pattern is divided into two layers, i.e., the central layer surrounded by creases B_1_B_2_, B_2_B_3_, B_3_B_4_, B_4_B_1_, and layer 1 enclosed between squares B_1_B_2_B_3_B_4_ and C_1_C_2_C_3_C_4_. Looking at the pattern from a different perspective, layer 1 can be considered as growing from the central layer through first extending crease A*_i_* B*_i_* (*i* = 1, 2, 3, 4) by the same length as theirs to generate creases B*_i_* C*_i_* (*i* = 1, 2, 3, 4) and then connecting vertex C_1_ with B_4_, C_2_ with B_1_, vertex C_3_ and B_2_, C_4_ with B_3_. Note that the entire pattern is still parameterized by a single geometric parameter, the side length of the square hub *a*. Subsequently, repeating the layer growth procedure as that for layer 1, new layers can be sequentially created, leading to extensible wrapping origami structures with any number of layers, which is recorded as *m*. The three‐layer and four‐layer patterns are respectively drawn in the middle and on the right of Figure 1B as examples, in which the central layer and layers 1, 2, and 3 are shown in blue, sapphire blue, light blue, and ice blue, respectively.

A physical specimen of the four‐layer design was fabricated, and folded by hand as shown in Figure 1C and Movie S1 (Supporting Information). The folding process is smooth from the initial state until noticeable resistance is encountered at state I. Upon further folding, the specimen is found to lock its shape at three states: II, III, and IV. Thus, the specimen appears to have quadra‐stability with four stable states, the initial state and states II to IV, which will be analyzed in detail below.

In addition to the square wrapping origami structures, those with any regular polygon with an even number of sides can be developed by the same method. Generally, to build a wrapping origami with an *n*‐side (*n* = 4, 6, 8…) regular polygonal central hub, new creases are required to create a regular polygon with *n*/2 sides in the central hub. An exception is when *n* = 4, a diagonal crease is needed. Then the pattern can be extended by introducing extra layers. A series of hexagonal^[^ ^61^ ^]^ and octagonal wrapping origami patterns are respectively shown in Figure 1D,F, and the folding processes of corresponding four‐layer physical specimens are respectively presented in Figure 1E,G and Movie S1 (Supporting Information). Both specimens are also found to have four stable states similar to their square counterpart.

### 2.2 Kinematics of the Extensible Wrapping Origami

**Figure 2:**
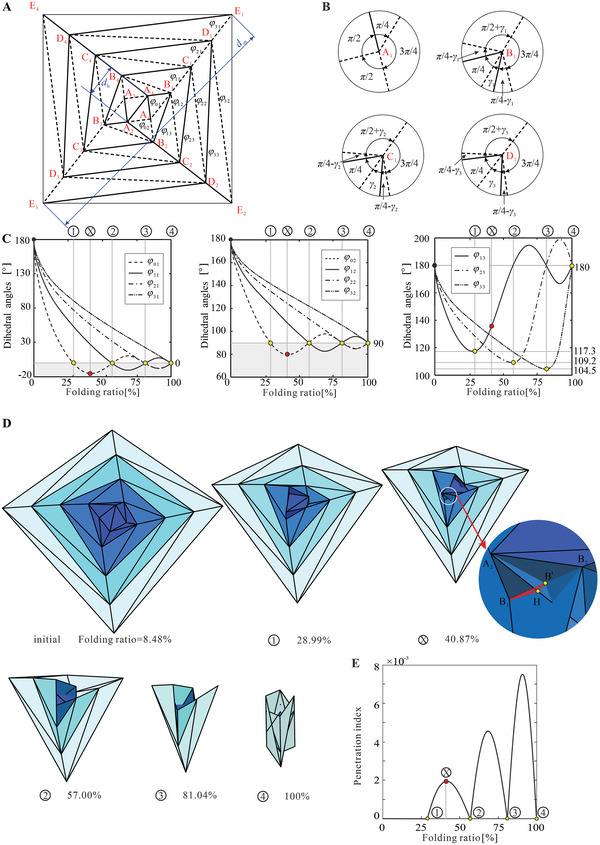
The kinematic analysis of the four‐layer square wrapping origami. A) The crease pattern of the four‐layer square wrapping origami with the identification of dihedral angles. B) Vertices A_1_, B_1_, C_1_, D_1_, and their surrounding creases. C) The kinematic relationships between the dihedral angle group one (φ_01_,φ_11_,φ_21_,φ_31_), two (φ_02_,φ_12_,φ_22_,φ_32_), and three (φ_13_,φ_23_,φ_33_) with the folding ratio respectively. D) The folding simulation of the four‐layer square wrapping origami. The state at folding ratio = 8.67% is the initial state. From the initial state to state ① is a rigid origami folding process. At state ①, the central layer has been tightly wrapped on the square hub; From state ①, the facet penetration occurs until the penetration disappears at state ②, where part of the creases of layer 1 pass through the facet of the central hub at state ⊗; The subsequent folding process is similar to the process from state ① to ②, the facet starts penetrating at state ② and disappears at state ③. Then facet starts penetrating at state ③ and disappears at state ④. E) Relationship between the penetration index and folding ratio.

To understand the folding mechanics and multi‐stable behaviors of the extensible wrapping origami, we first investigated its kinematics. Taking the four‐layer square wrapping origami as an example, its denotations of vertices and dihedral angles between two facets joining at one crease are shown in **Figure** **2**A. By treating the creases as revolute joints and the facets as links, the crease pattern was modeled as a network of spherical linkage. Then the linkage network transformed to the truss form (Figure S1A,B, Supporting Information), and its kinematic properties were analyzed by the truss method,^[^ ^62^ ^]^ details in Supporting Information. Note that facet penetration was allowed in the analysis to manifest the differences in motion among different layers. The analysis results show that the structure has a single degree of freedom. Due to the four‐fold rotational symmetry of the pattern except for the central hub, only vertices A_1_, B_1_, C_1_, and D_1_, respectively located in the central layer and layers 1, 2, and 3, need to be analyzed. The sector angles at the four vertices are shown in Figure 2B, where γ_1_ =  arctan(1/3) at vertex B_1_, γ_2_ =  arctan(3/5) at vertex C_1_, γ_3_ =  arctan(5/7) at vertex D_1_. Subsequently, three groups of dihedral angles categorized by their positions in each layer, φ_01_, φ_11_, φ_21_, φ_31_ in group one, φ_02_, φ_12_, φ_22_, φ_32_ in group two, φ_13_, φ_33_, φ_33_ in group three, are plotted in Figure 2C with respect to the folding ratio. Here the folding ratio of the structure is defined as (*d*_m_ *‐d*)/(*d*_m_ *‐d*_h_), where *d*_m_ = $\sqrt{130}a\;$is the distance between the two diagonal vertices of the structure in the fully deployed state as shown in Figure 2A, *d* is the distance between these two points in any state, and *d*_h_ $=\sqrt{2}\;a$ is the diagonal of the central hub.

The folding process of the structure can be illustrated by the variations of the dihedral angles in group one presented in the left subfigure of Figure 2C. In the beginning, the folding of the structure is a rigid origami process. All four angles decrease during folding but at different rates. At state ① with a folding ratio of 28.99%, φ_01_ is first reduced to 0°, indicating that the central layer has been tightly wrapped around the central hub as shown in the corresponding configuration in Figure 2D and Movie S2 (Supporting Information). At this time, the other three dihedral angles φ_11_, φ_21_, and φ_31_, respectively located in layers 1, 2, and 3, lag behind φ_01_, with φ_11_= 53.8° < φ_21_= 77.8° < φ_31_= 91.3°, and therefore all those layers are still partially folded. As the structure is further folded, φ_01_ becomes negative and enters the gray area, suggesting that interference among the facets occurs in the central layer and thus the folding is physically a non‐rigid origami process. Moreover, the magnitude of φ_01_ first decreases, reaching its minimum at state ⊗, and then gradually rises. In contrast, the other three layers are still folded in a rigid origami manner as φ_11_, φ_21_ and φ_31_ remain positive. When the structure reaches state ② (folding ratio = 57.00%), φ_11_ also reaches 0° ahead of φ_21_ and φ_31_, with φ_21_ < φ_31_. This means that both the central layer and layer 1 are fully wrapped, but layers 2 and 3 are still a distance from the central hub, see configuration ② in Figure 2D. Meanwhile, φ_01_ returns to 0°, indicating that facets penetration disappears. In other words, the entire structure is in a rigid origami state. Further folding the structure, a similar sequential wrapping process is observed. From state ② to ③ (folding ratio from 57.00% to 81.04%), layer 2 is fully wrapped, while layer 3 is still open, and from state ③ to ④ (folding ratio from 81.04% to 100%), layer 3 is finally fully folded. Therefore, the folding process of the four‐layer square structure is characterized by a rigid origami range and three rigid origami states, separated by three non‐rigid origami folding ranges. Such feature is also supported by the variation of φ_02_,φ_12_,φ_22_,φ_32_ in group two, and φ_13_,φ_33_,φ_33_ in group three, which are also depicted in Figure 2C. For the dihedral angle in group two, since the central hub is square, the minimum dihedral angle wrapped around the central hub is 90° and facet penetration occurs when they are less than 90°. Similarly, when the dihedral angles in group three reach their minimum values 104.5°,109.2°, and 117.3°, respectively, the corresponding creases contact the central hub and then facet penetration occurs. At state ⊗ in Figure 2D, the crease B_1_B_2_ corresponding to φ_13_ passes through the central hub.

The unique sequential folding feature and the existence of rigid origami states, which will be later shown to be critical for the multi‐stability of the extensible wrapping origami, are determined by the crease arrangement and sector angles at vertices A_1_, B_1_, C_1_, and D_1_. Specifically, the central layer is fully wrapped ahead of the others because of the fewer creases at vertex A_1_ compared to B_1_, C_1_, and D_1_. Moreover, as shown in Figure 2B, the sequential folding process of layers 1 to 3 is attributed to the monotonically increasing sector angles, i.e., γ_1_ < γ_2_ < γ_3_, which can be calculated as tan γ_*i*_ = (2*i* − 1)/(2*i* + 1) , where *i* is the serial number of the layer. Without such a folding feature, simply increasing the number of layers does not lead to multi‐stability. For example, adding more layers to the bistable square origami hypar^[^ ^51^ ^]^ cannot bring more stable states, but only affects the force versus displacement response of the structure. In addition, the transition among different stable states of the proposed structure can be achieved simply by a uniaxial loading, thus avoiding the sophisticated deformation control at the bifurcation point for the multi‐stable hexagonal hypar.^[^ ^57^ ^]^

A non‐dimensional penetration index, defined as the total area penetrated by the creases dividing that of the circumscribed circle of the structure in the fully deployed state,$\;2nS_{\Delta }/\left(\pi d_{m}^{2}\right)$ is introduced to quantify the amount of fictional facet penetration during non‐rigid origami folding. Taking the state ⊗ in Figure 2D as an instance, as shown in the partially enlarged view, crease B_1_B_2_ passes through the central hub and intersects at point B', and point H is the intersection point by drawing a perpendicular line from point B' to crease A_1_A_2_. The area penetrated by crease B_1_B' when it passes through the hub is triangle B_1_B'H, i.e., *S*_Δ_. Combined with the Truss method, the coordinates of each vertex in the folding process can be obtained, and then the geometric information of penetration crease B_1_B' and penetration area triangle B_1_B'H can be obtained. Thus, the penetration index of the above four‐layer structure is calculated and drawn in Figure 2E. It can be seen that this index is zero in the rigid origami range and states, whereas forming three peaks in the non‐rigid origami ranges. In addition, the maximum values of the three peaks, which are corresponding to the facet penetration from layer 1 to layer 3, show a trend of monotonic increase, suggesting higher energy barriers are required to surpass when the structure is wrapping from inner to outer layers.

Finally, the kinematic curves of the two‐layer and three‐layer square wrapping origami are also obtained by the above method, and are compared with those of the four‐layer square wrapping origami. As shown in Figure S1C–E (Supporting Information), the kinematic properties of the same layer of the three structures are identical, in other words, the four‐layer square wrapping origami can show the kinematic properties of the two‐layer and three‐layer ones. Detailed analysis can be found in Supporting Information. This conclusion can be extended to the wrapping origami with other even‐numbered regular polygons as hubs and with more layers, provided that the rotational symmetry of the wrapping origami is maintained so that it has a single degree of freedom during folding. This is because, for the wrapping origami with even‐sided hubs, the introduction of extra layers will not alter the pattern of the existing layers. As long as the folding of the structure is limited to a single degree of freedom, the wrapping kinematic properties of the existing layers remain unaffected by the newly introduced layers.

### 2.3 Deformation and Multi‐Stable Behavior of the Extensible Wrapping Origami

Having understood the sequential folding kinematics of the extensible wrapping origami patterns, here we study the deformation mechanics and multi‐stable behavior through experiments and numerical simulation. Physical specimens of the two‐layer, three‐layer, and four‐layer square wrapping origami structures with side length *a* = 30 mm and panel thickness *t* = 0.5 mm were manufactured from PET and reinforced at panels by 304 stainless steel sheets, and then folded quasi‐statically by a universal testing machine INSTRON 6800. The detailed manufacturing method and experimental setup can be found in Supporting Information.

**Figure 3:**
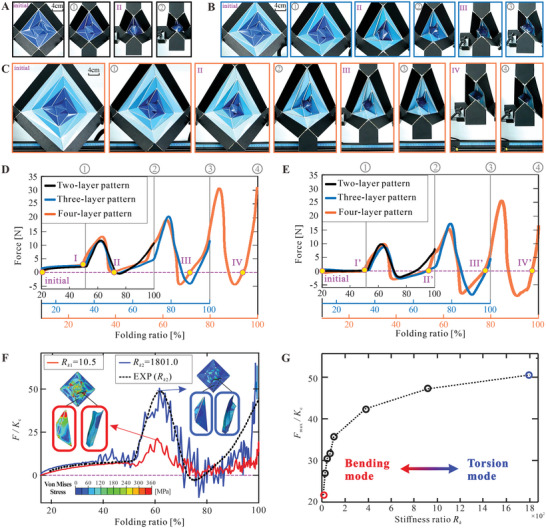
Deformation and multi‐stable behavior of the square wrapping structures. A–C) The experimental folding process of the two‐layer, three‐layer, and four‐layer square wrapping origami structures respectively. Configuration I is when the force rises sharply, and II, III, and IV are stable states. Configurations ①, ②, ③, and ④ are the states in Figure 2D. D) The experimental force versus folding ratio curves of the two‐layer, three‐layer, and four‐layer square wrapping origami structures. The folding ratios of the three structures are aligned by kinematic results in Figure 2C. E) The amended force versus folding ratio curves after deducting the contribution of the creases. F) The normalized force versus folding ratio curves of the two‐layer square wrapping origami under different stiffness ratios: simulation result under the smallest stiffness ratio (*R*_k1_ = 10.5), simulation and experimental result under the largest stiffness ratio (*R*_k2_ = 1801.0). The characteristic deformation modes of highlighted trapezoidal region comprising two triangular panels and one crease under the two stiffness ratios are also shown. G) The normalized local peak forces versus stiffness ratio curve. The gradient color from red to blue represents the deformation transition from bending mode to torsion mode in Figure 3F.

The folding processes of these three structures with two, three, and four layers are respectively presented in **Figure** **3**A–C and Movie S3 (Supporting Information), and the force versus folding ratio curves are shown in Figure 3D. The configurations in Figure 3A–C correspond to the stable states with zero force (initial, II, III, and IV, noted as yellow circles in Figure 3D) and the states ① to ④ in Figure 2D (marked by solid gray lines in Figure 3D). As expected, the three structures respectively show bistability, tri‐stability, and quadra‐stability. Moreover, both the folding modes and force curves of the corresponding layers in the three structures are very similar, i.e., the four‐layer structure reproduces the behavior of the two‐layer and three‐layer ones while exhibiting the unique folding behavior of layer 3. This observation indicates that the introduction of new layers does not affect the behaviors of existing ones. And since the pattern is further extendable, it can be reasonably deduced that every extra layer will bring one more stable state.

Subsequently, the four‐layer structure is further analyzed as a representative. It can be seen from the force versus folding ratio curve that from the initial state to configuration ① that corresponds to the rigid origami folding range predicted by the kinematic analysis, the central layer is gradually folded, and the force increases very mildly to overcome the torsional stiffness of the creases. As the folding further progresses, the force quickly rises due to panel interference in the central layer and then snaps through to the second stable state II. At this time, layer 1 is not fully wrapped until it reaches configuration ②. It should be mentioned that according to the kinematic analysis, the physical interference in the central layer disappears at configuration ②. If the creases are ideal hinges with zero stiffness, configuration ② should be a stable state as there is zero energy in the system. The phase shift between the theoretical and experimental stable states is attributed to the finite torsional stiffness of the creases. Corresponding, by assuming that the creases have an elastic perfectly plastic moment versus rotation relationship and rotate following the kinematic curves in Figure 2C, the contribution of the creases can be deducted from the reaction force, see more details in Figure S5 (Supporting Information). The amended force versus folding ratio curves in Figure 3E shows that the new stable state II' is very close to the theoretical configuration ②. Subsequently, layer 2 and layer 3 are sequentially folded, creating two more stable states III and IV that can be modified in the same manner to match the corresponding theoretical ones. Also note that the local peak forces increase layer by layer, which is in accordance with the trend in the penetration index shown in Figure 2E. To summarize, by jointly considering the kinematics and creases properties, the deformation mode and positions of stable states can be accurately predicted.

Finally, while achieving the same global multi‐stability, the extensible wrapping structures exhibit different local deformation mechanisms depending on the ratio of panel stiffness to crease stiffness, denoted as *R*_k_ hereafter, the calculation of which is given in Supporting Information. This was investigated through finite element analysis with Abaqus/Explicit, the detailed setup of which is provided in Figure S6 (Supporting Information). The numerical counterparts for all three physical specimens in Figure 3A–C were built, analyzed, and compared with the experimental data. In general, a good agreement in terms of folding mode and reaction force is achieved, see more details in Figure S6B–D (Supporting Information), and the results of the two‐layer structure are shown in Figure 3F as a representative. Then a series of numerical specimens with identical two‐layer geometry and crease stiffness but varying panel stiffness from 22 to 3782 N rad^−1^ are analyzed. Comparing the specimen with the smallest stiffness ratio *R*_k_ of 10.5 and that with the largest ratio of 1801.0, it is found from Figure 3F that in the rigid origami range where crease rotation dominates, the two force curves are very close. As they enter the non‐rigid range, the specimen with a large stiffness ratio has a much larger local peak force. Moreover, the von Mises stress contours of the two specimens corresponding to their respective local peak forces are also presented in Figure 3F. Under both stiffness ratios, the deformation is mainly concentrated in the highlighted trapezoidal region comprising two triangular panels and one crease. However, when the ratio is small, the main deformation mechanism is the bending of the triangular panels. When the ratio is large, noticeable crease torsion is observed as it becomes too difficult to bend the panels. See Figures S7 and S8 (Supporting Information) for more results on deformation mechanisms. The local peak forces normalized by the crease stiffness are drawn against the stiffness ratio in Figure 3G. With the increase in stiffness ratio, the local peak force also rises, indicating a more prominent snap‐through bistability behavior. And the local deformation mechanism gradually transforms from the bending mode in red to the torsion mode in blue. It is also worth noting that when the stiffness ratio is further reduced, the local peak as well as the second stable state will disappear. Thus, there exists a lower bound of the stiffness ratio to achieve multi‐stability.

**Figure 4:**
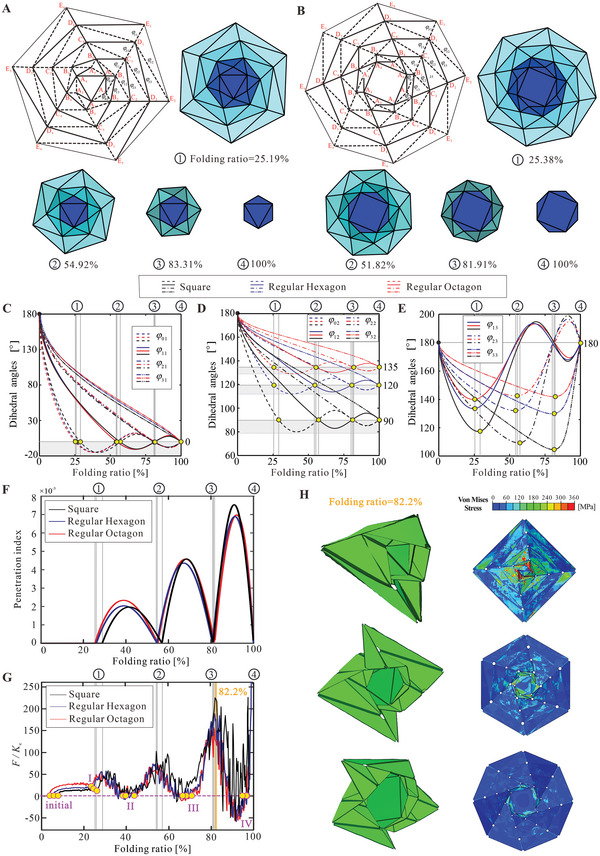
Multi‐stable behavior of the extensible origami structures with different hub shapes. A,B) The crease pattern and folding simulation of the four‐layer hexagonal and octagonal wrapping origami structures, respectively. At state ①, the central layer has been tightly wrapped on the square hub and some panel contact; At states ②, ③, and ④, the structure has no interference. C–E) The kinematic relationships of the dihedral angles of the four‐layer square, hexagonal, and octagonal structures, taking the folding ratio as input. F) Relationship between the penetration index and folding ratio of the four‐layer wrapping structures. G) The normalized force versus folding ratio curves of the four‐layer square, hexagonal, and octagonal structures. The rigid origami states in Figures 4A,B and 2D, as well as the stable states in Figures 1C,E,G, are also marked. H) The deformation and the von Mises contours project back to the initial states of the three structures at the folding ratio of 82.2% corresponding to the highest local peaks.

The multi‐stability is not a unique characteristic of the square wrapping structures, but an intrinsic feature for this kind of extensible wrapping origami. To demonstrate this, a four‐layer hexagonal structure and a four‐layer octagonal structure, which can reproduce the folding behaviors of the corresponding two‐layer and three‐layer ones in Figure 1D,F, are respectively presented in **Figure** **4**A,B. The origami structures have identical circumscribed circle diameters with that for square one and follow the same naming conventions for vertices and dihedral angles, which are analyzed using the Truss method. These two structures do not possess a single degree of freedom in the rigid origami range. Therefore, additional constraints are introduced to maintain rotational symmetry during the folding process. Detailed calculation procedures and results, as well as the kinematic curves of the two‐layer and three‐layer wrapping origami, can be found in Figures S9–S11 (Supporting Information). The folding process of the two structures are respectively shown in Figure 4A,B, and the same three groups of dihedral angles of the two structures are respectively drawn in Figure 4C–E against the folding ratio, together with those of the four‐layer square one previously studied in Figure 2C. These kinematic curves show that the three extensible wrapping origami patterns have the same sequential folding feature, and the distribution of rigid origami states is also very similar. The partially enlarged view of panel penetration of the four‐layer hexagonal structure is shown in Figure S12 (Supporting Information), which is very similar to that of the four‐layer square structure. And the penetration indices of the three structures shown in Figure 4F are found to be very close.

Subsequently, the quasi‐static folding behaviors of the hexagonal and octagonal wrapping structures are simulated using the same materials and setup as those for the square ones (details in Figure S6, Supporting Information), and the normalized force versus folding ratio curves are drawn in Figure 4G together with that of the square structure in Figure S6D. Not surprisingly, all three structures exhibit quadra‐stability and reach stable states at initial, II, III, and IV states. The octagonal structure has the largest force in the beginning rigid origami range since it has the most creases. The local peak forces that are mainly determined by the penetration index are very close among different structures. However, the von Mises stress contours of the three structures at the folding ratio of 82.2% corresponding to the highest local peaks, which are presented in the undeformed configurations in Figure 4H, indicates that the maximum stress in the octagonal structure is 87.5% lower than that of the square one. Therefore, despite not being able to tune the magnitude of local peaks, increasing the number of sides helps to reduce the maximum stress in the structure to achieve a more uniform stress distribution, thus avoiding or mitigating material failure during the folding process. Together with the stiffness ratio, this will enable programmability of the structure properties through geometric parameters and base materials.

## 3 Discussion

To summarize, through modification of the original non‐rigid foldable wrapping origami pattern and extending it by adding additional circumferential layers, we have designed a series of square, hexagonal, and octagonal extensible origami structures with generic multi‐stability. Through a kinematic analysis with the truss method, we have found out that a sequential folding process from the central layer to the extended ones of the structure is created, thereby generating a continuous rigid origami range and several discrete rigid origami states separated by non‐rigid foldable ranges. And the number of rigid origami states is determined by that of the extended circumferential layers. Subsequently, we have fabricated physical samples of square extensible structures and conducted uniaxial compression experiments, from which the multi‐stable behaviors of the structures are confirmed. By eliminating the effect of crease stiffness, the stable states of the structures match the rigid origami states reasonably well, thus proving the critical role of the pattern folding mechanics in creating the multi‐stability of the structure. Finally, the effects of material and geometric parameters of the structure on the mechanical properties are investigated through numerical simulation. It is found that a large panel stiffness to crease stiffness ratio leads to noticeable crease torsion and a more prominent snap‐through phenomenon, while a small ratio causes the formation of traveling hinges in the panels and lower local peak forces. Moreover, increasing the number of sides of the structure has little effect on the magnitude of local peak forces, but generates a more uniform stress distribution in the structure. Although a maximum of four layers in a structure is considered in the work, leading to quadra‐stability, the proposed structure can be readily extended further to achieve more stable states. In addition, the proposed structure can be tessellated to form larger ones as shown in Figure S13 (Supporting Information). Altogether, this work offers a distinctive and effective design approach for generic multi‐stable structures. In the future, we will employ the design principle in this work to develop multi‐stable structures out of other non‐rigid foldable origami patterns. Moreover, Due to the fact that design of the crease lines is critical for the mechanical performance and fatigue life of origami structures under the repeating folding/unfolding process,^[^ ^63^ ^]^ we will explore the optimal crease design for the structures to achieve the robust multi‐stability with a long fatigue life. Finally, efforts will be made to incorporate the multi‐stable structures into advanced engineering applications such as soft robotics, mechanical computing, and multi‐functional metamaterials.

## 4 Experimental Section

### Manufacture of Physical Specimens

The square wrapping origami specimens were manufactured with a 0.1 mm PET sheet in the middle and two 0.2 mm 304 stainless steel sheets on both sides, refer to Figure S2D (Supporting Information). The material properties of PET were characterized through tensile testing, with the main mechanical properties listed in Table S3 (Supporting Information). Thin films with gradually lighter colors from the inner layer to the outer layer were tied on one side of the specimens. To eliminate the prestressing effect of manual folding, heat treatment was also executed. The specimens were fixed at the initial state and heated in a constant temperature furnace at 55 °C for 1 h. More details about the design of the patterns with creases offset and the manufacture of physical specimens are provided in the Supporting Information.

### Experimental Setup

Folding experiments were conducted to explore multi‐stable behaviors. A series of boundary frames were first designed as illustrated in Figure S2E (Supporting Information). The frame folds with a single degree of freedom when only the horizontal displacement was enabled on both loading ends. The frame panels were made of 2 mm stainless steel and linked by hinges. Fiber tape was employed to connect the frames and the three specimens, as shown in Figure S2F (Supporting Information). The setup of the folding experiment by the INSTRON 6800 testing machine for horizontal loading with a speed of 50 mm min^−1^ is shown in Figure S2G (Supporting Information). By removing the force data of the frame‐only loading in Figure S2H (Supporting Information), the influence of frame gravity was modified for each experimental loading. More details about the experimental setup and numerical simulation setup are provided in the Supporting Information.

### Statistical Analysis

The experimental results were derived from reproducible experimental data obtained after finite preloading (the fourth experimental loading). The normalization of force data is described in the Supporting Information. Statistical analysis was conducted using Microsoft Excel 2021 and Matlab_R2020b.

## Conflict of Interest

The authors declare no conflict of interests.

## Author Contributions

K.X. and S.C. contributed equally to this work. All the authors conceived the work. K.X. and S.C. performed a literature survey. J.M., S.C., and K.X. wrote the summary and conclusion of the manuscript. K.X. performed the kinematics analysis. S.C. performed experiments and simulations. K.X. drafted the description of the geometric design and kinematics. S.C. drafted the description of the deformation and multi‐stable behavior. J.M. and Y.C. revised the manuscript. All authors contributed to the discussion and manuscript review.

## Supporting information

Supporting Information

Supplemental Movie1

Supplemental Movie2

Supplemental Movie3

## Data Availability

The data that support the findings of this study are available from the corresponding author upon reasonable request.
