# Supplementary material for: Multi‐Stability of the Extensible Origami Structures
Source: Adv Sci (Weinh). 2023 Aug 8;10(29):2303454. doi: 10.1002/advs.202303454 (PMC10582408; doi:10.1002/advs.202303454)
Supplement: Supplementary file 1 — Supporting Information [file ADVS-10-2303454-s002.pdf]

ADVANCED SCIENCE
Open Access

Supporting Information

for *Adv. Sci.*, DOI 10.1002/advs.202303454

Multi-Stability of the Extensible Origami Structures

Kaili Xi, Sibo Chai, Jiayao Ma and Yan Chen**

Supporting Information

Multi-Stability of the Extensible Origami Structures

Kaili Xi, Sibo Chai, Jiayao Ma, and Yan Chen**

K. Xi, S. Chai, J. Ma, Y. Chen
Key Laboratory of Mechanism Theory and Equipment Design of Ministry of Education, Tianjin University, 135 Yaguan Road, Tianjin, 300350, China.
School of Mechanical Engineering, Tianjin University, 135 Yaguan Road, Tianjin, 300350, China.

K. Xi and S. Chai contributed equally to this work.
E-mail: yan_chen@tju.edu.cn (Y. Chen), jiayao.ma@tju.edu.cn (J. Ma).

This PDF file includes:

1. The Kinematic Analysis of the Square Wrapping Origami
2. Manufacture of Physical Specimens
3. Experimental Setup
4. The Energy and Force Caused by Crease Folding
5. Bending Stiffness of Panels and the Stiffness Ratio
6. Numerical Simulation Setup
7. Deformation Mechanisms under Different Stiffness Ratios
8. Kinematic Analysis of the Hexagonal and Octagonal Wrapping Origami
9. Tessellation of the wrapping origami
10. Figures S1 to S13
11. Tables S1 to S5
12. Legends for Movies S1 to S3
13. References

1. The Kinematic Analysis of the Square Wrapping Origami

The truss form of the four-layer square wrapping origami includes bars of b = 53 and nodes of j = 20 as shown in Figure S1A. The geometric dimension is a = 30 mm. Figure S1B shows the general unfolded state and Cartesian coordinate system. The origin O is below the whole model, and the z-axis is perpendicular to plane $A_4A_1A_3$. The coordinates of vertices can be obtained according to the system, as expressed in Table S1. The equilibrium equations of the truss composed of b bars and j nodes are as follows:

$$At = f \tag{S1}$$

where A is a $3j\times b$ equilibrium matrix, t is a $b\times 1$ vector of bar axial forces per unit length, and f is a $3j\times 1$ vector of node forces. Since the external forces are ignored in the Truss, Eq. (S1) becomes

$$At = 0 \tag{S2}$$

The rank of equilibrium matrix A is designed as r, so the number of self-stresses can be obtained from

$$s = b - r \tag{S3}$$

and the number of mobility can be derived from

$$m = 3j - r - 6 \tag{S4}$$

The equilibrium matrix A can be established according to the Truss Transformation method, and the result is the rank r = 53, the numbers of self-stresses s = 0, and mobility m = 1. Thus, the four-layer wrapping origami pattern is a one-DOF mechanism without constraint. The same analysis applies to the two-layer and three-layer wrapping origami, and the kinematic curves of the two-, three-, and four-layer square wrapping origami can be obtained by the Truss method. Figure S1C shows the comparison of the relations between dihedral angles and the input dihedral angle φ_{04}, which is located in the central layer of the two-, three-, and four-layer square wrapping origami, respectively. It can be seen that the above three groups of kinematic curves are completely coincident, which means that the kinematics properties of the central layer of these structures are the same. Next, Figure S1D shows the comparison of the relations between dihedral angles $\varphi_{11}, \varphi_{12}, \varphi_{13}$, which are located in layer 1 of the two-, three-, and four-layer square wrapping origami, and the folding ratio of the two-layer one. Besides, Figure S1E shows the comparison of the relations between dihedral angles $\varphi_{21}, \varphi_{22}, \varphi_{23}$, which are located in layer 2 of the three-layer and four-layer square wrapping origami, and the folding ratio of the three-layer one. These results show that the kinematic curves of the same layers of the two-, three-, and four-layer square wrapping origami are identical, in other words, the four-layer

square wrapping origami can reproduce the kinematic properties of two-layer and three-layer ones.

2. Manufacture of Physical Specimens

The multi-stable characteristics of the structures can be only verified from the folding process of the physical specimens. A common manufacturing obstacle of the wrapping origami structures is the significant increase in the cylinder hub's radius with the accumulation of wrapped material, leading to thickness interference. This interference will trigger erratic deformation modes and deviate from the kinematics of the aimed wrapping mode. To address this issue, some of the creases need to be offset. [1,2] On this basis, specimens of two-layer, three-layer, and four-layer square wrapping patterns were manufactured in the form of stacked panels with membrane backing. [3] Figure S2A shows the geometries of the flattened state and tightly wrapped state. The black half represents the kinematic model with zero thickness, while the blue half represents the physical specimen with a definite thickness of t. The offset distances δ caused by thickness accumulation were adopted to eliminate interference, applying on the final edges of the wrapping hub, i.e., A_1B_2, B_1C_2, C_1D_2, D_1E_2, and other creases obtained by four-fold rotational symmetry in Figure 2A. The offset distances δ, shown in Figure S2B, are t (A_1B_2), $2t$ (B_1C_2), $3t$ (C_1D_2), and $4t$ (D_1E_2) from the inside to the outside. The original crease defines the boundary of the inner panel, while the offset crease defines the boundary of the outer panel. The region between the original crease and offset crease is regarded as the wide crease region with δ. The distance between the offset edge of the inner crease and the original edge of the adjacent outer crease remains a constant length a, as presented in Figure S2A. In addition, all the crease also needs to have a width to fold easily. Considering that the maximum folding angle of the creases is 180°, the crease width should be equal to the thickness t. Therefore, each crease was further offset by a distance η on both sides, where $\eta=t/2$. The offset η doesn't change the position of the creases but only widens the creases. For the creases that already have δ offsets, the η offsets were also applied to both sides of the wide crease, resulting in the final widths of all creases being either 2η or $\delta+2\eta$. Moreover, a circular hole with a radius of r was set at the intersection of creases to avoid stress concentration. Here, the radius r from inside to outside is $3t$ (vertex A_i, i =1, 2, 3, 4), $4t$ (vertex B_i), $5t$ (vertex C_i), and $6t$ (vertex D_i), all of which are provided in Figure S2B.

Moreover, the offset δ influences the overall dimension and folding displacement of the wrapping origami structures, which has been modified further. With the side length of the

square hub *a* set to 30mm and the panel panel thickness *t* set to 0.5mm, the wrapping pattern geometry can be completely determined. The distance between the two diagonal vertices *d* is taken as the input parameter. The distances of the flattened state and tightly wrapping state of the kinematic models (d_m and d_h) and the thickness-offset physical specimens (d_m' and d_h') are listed in Table S2. The distance at other physical loading positions, d', can be obtained by projecting the offset region to the horizontal plane shown in Figure S2C using equation (S5).

$$d' = d + 2\sum_{i=1}^{m} \delta_i \frac{\cos\alpha_i}{\cos\beta_i} \tag{S5}$$

Where δ_i is the offset width of *i* th layer, $\boldsymbol{n_i}$ is the normal vector of the *i* th (i = 4, 6, 8...) offset crease in the located surface of the panel, $\boldsymbol{m_i}$ is the projection vector of $\boldsymbol{n_i}$ to the horizontal plane, α_i is the spatial angle between $\boldsymbol{m_i}$ and $\boldsymbol{n_i}$, and β_i is the angle between $\boldsymbol{m_i}$ and the direction of the two diagonal vertices of the structure. In addition, an initial folding angle is necessary to fold the origami pattern from the flattened state. The state with the largest dihedral angle of A_2A_4 is set as the initial state (refer to Figure S1C). At this initial state, the loading distances of the zero-thickness models and the thickness-offset specimens are d_0 and d_0', respectively, listed in Table S2. The length of the loading stroke can consequently be calculated as d_0'-d_h'.

The square wrapping origami specimens were manufactured with a 0.1 mm PET sheet in the middle and two 0.2 mm 304 stainless steel sheets on both sides, refer to Figure S2D. The wide crease region comprises only the 0.1 mm PET sheet, while the panel area is composed of 0.5 mm stacked materials, resulting in significant differences in stiffness. The material properties of PET were characterized through tensile testing, with the main mechanical properties of density ρ, Young's modulus E, and yield stress σ_s listed in Table S3. The material tensile curve of PET is displayed in Figure S3. The loading-unloading experiment, as shown in Figure S3A, was conducted to measure the residual strain under various loading strains. The loading point corresponding to 0.2% residual strain is taken as the yield one, which is also marked on the entire tensile curve in Figure S3B. Thin films with gradually lighter colors from the inner layer to the outer layer were tied on one side of the specimens. To eliminate the prestressing effect of manual folding, heat treatment was also executed. The specimens were fixed at their initial state and heated in a constant temperature furnace at 55 ℃ for 1 h.

3. Experimental Setup

Further, folding experiments were constructed on the specimens to explore the multi-stable behaviors. A series of boundary frames were first designed as illustrated in Figure S2E. The frame folds with a single degree of freedom with the horizontal displacement was constrained on both loading ends. Kinematic analysis has revealed that the combination of frames does not change the folding behaviors of the wrapping origami structure. Therefore, the frame can serve a dual role of both boundary strengthening and loading. The frame panels are made of 2 mm stainless steel and linked by hinges. Fiber tape is employed to connect the frames and the three specimens, as shown in Figure S2F. The setup of the folding experiment by the INSTRON 6800 testing machine for horizontal loading with a speed of 50 mm min^{-1} is shown in Figure S2G. By removing the force data of the frame-only loading in Figure S2H, the influence of frame gravity was modified for each experimental loading.

In addition, the gravity of the specimens also does a great deal of work. It is found that whether the specimens or the loading frame, the gravity center is always the geometric center of the structure, as the height h can be defined by

$$h = \sqrt{\frac{{l_m}^2}{4} - \frac{(d_m - d)^2}{8}} = \frac{1}{2}\sqrt{(4m^2+1)a^2 - \frac{(d_m - d)^2}{2}} \tag{S6}$$

where l_m is the length of the boundary side, and m is the layer number of the specimens. Then the loading force of the gravity component can be obtained:

$$F_G = G\frac{dh}{dx} = G\frac{\sqrt{2}l - d}{2\sqrt{4\sqrt{2}ld - 2d^2}} \tag{S7}$$

This equation is suitable for both the specimen and the frame. The mass attributes of each structure are listed in Table S4. The experimental force versus folding ratio curves before and after removing the gravity influence of wrapping origami structures is illustrated in Figures S4A, 3D respectively. The modified results of the frame are also analyzed and compared with the curve under frame-only loading in Figure S4B. The force results correspond perfectly, which verifies the modification method.

4. The Energy and Force Caused by Crease Folding

With the experimental parameters of the wrapping origami structures, the energy of crease folding cannot be ignored. We assume that the creases have an elastic perfectly-plastic moment versus rotation relationship. As shown in Figure S5A, we defined M as the bending moment

per unit length, K_c as the torsional elastic constant per unit length in the elastic phase, and M_c as the perfect plastic bending moment per unit length in the plastic phase. Subsequently, the folding energy and folding force can be calculated as follows

$$U_{\text{fold}} = \begin{cases} \sum_{i=1}^{n_{\text{crease}}} K_c l_i (\varphi_i - \varphi_{i,0})^2 / 2, & \varphi_i < \varphi_{i,0} + \Delta\varphi_y \\ \sum_{i=1}^{n_{\text{crease}}} M_c l_i (\varphi_i - \varphi_{i,0} - \Delta\varphi_y / 2), & \varphi_i \geq \varphi_{i,0} + \Delta\varphi_y \end{cases} \quad \text{(S8)}$$

$$F_{\text{fold}} = \partial E_{\text{fold}} / \partial u \quad \text{(S9)}$$

where U_{fold} is the energy of crease folding, n_{crease} is the number of all creases, l_i is the length of i th crease, φ_i is the dihedral angle of i th crease, $\varphi_{0,i}$ is the dihedral angle of i th crease at the initial state, $\Delta\varphi_y$ is the range of dihedral angle variation before the yield point, F_{fold} is the loading force caused by crease folding, and u is the displacement of the loading ends. It should be noted that some dihedral angles in Figure 2C have intervals with reverse folding. Therefore, the energy of crease folding must be calculated in segments, i.e., the turning point is defined as $\varphi_{0,i}'$. Through the experimental data of the two-layer square wrapping structure, K_c and M_c are fitted as K_c=2.1 N rad^{-1} and M_c = 1.9 N, as shown in Figures S5A,B. Then the folding energy and folding force curves of the three structures are shown in Figures S5C,D. Noted that the normalization of force in this work is carried out through K_c.

5. Bending Stiffness of Panels and the Stiffness Ratio

The stiffness ratio is changed by keeping the thickness of the PET sheet (t_{pet}) constant and continuously reducing the thickness of the steel sheet (t_{steel}). The stiffness ratio R_k is defined as the ratio of the panel's torsional elastic constant per unit length of panel K_p to the crease's torsional elastic constant per unit length K_c. The panel is a laminated composite structure of steel and PET as shown in Figure S2D and its stiffness can be calculated as follows

$$\frac{1}{\rho} = \frac{M}{EI} = \frac{M_1 + M_2 + M_3}{EI} \quad \text{(S10)}$$

$$\frac{1}{\rho_1} = \frac{M_1}{E_1 I_1} \quad \frac{1}{\rho_2} = \frac{M_2}{E_2 I_2} \quad \frac{1}{\rho_3} = \frac{M_3}{E_3 I_3} \quad \text{(S11)}$$

The subscript numbers 1, 2, and 3 refer to the upper steel sheet, the central PET sheet, and the lower steel sheet respectively. Since the thickness is quite small relative to the length dimension, the curvature of each layer can be approximated to the curvature of the stack

$$\rho_1 = \rho_2 = \rho_3 = \rho \quad \text{(S12)}$$

The elastic modulus and moment of inertia of each sheet are as follows

$$E_1 = E_3 = E_{\text{steel}} \tag{S13}$$

$$E_2 = E_{\text{pet}} \tag{S14}$$

$$I_1 = I_3 = \frac{l t_{\text{steel}}{}^3}{12} + \frac{l t_{\text{steel}}}{4}(t_{\text{steel}} + t_{\text{pet}})^2 \tag{S15}$$

$$I_2 = \frac{l t_{\text{pet}}{}^3}{12} \tag{S16}$$

Then the torsional elastic constant per unit length of the laminated panel can be obtained

$$K_{\text{p}} = \frac{E_1 I_1 + E_2 I_2 + E_3 I_3}{ls} = \frac{2E_{\text{steel}} t_{\text{steel}}{}^3 + 6E_{\text{steel}} t_{\text{steel}}(t_{\text{steel}} + t_{\text{pet}})^2 + E_{\text{pet}} t_{\text{pet}}{}^3}{12s} \tag{S17}$$

where s is the length of the bending arc. To unify parameters, the crease width of 2η was selected as the bending arc length of panels to calculate the stiffness ratio by assuming that the creases are uniformly bending in the width direction. Finally, the stiffness ratio of the wrapping structure is as follows:

$$R_k = \frac{K_{\text{p}}}{K_{\text{c}}} = \frac{2E_{\text{steel}} t_{steel}{}^3 + 6E_{\text{steel}} t_{\text{steel}}(t_{\text{steel}} + t_{\text{pet}})^2 + E_{\text{pet}} t_{\text{pet}}{}^3}{24\eta K_{\text{c}}} \tag{S18}$$

The torsional elastic constant per unit length of creases is given as K_{c}=2.1 N rad^{-1}. The panel thickness, panel stiffness, and corresponding stiffness ratio selected in this work are shown in Table S5. In particular, the smallest stiffness ratio is R_{k1}=10.5, and the largest stiffness ratio is R_{k2}=1801.0. Among them, R_{k2} is also the stiffness ratio of the experimental specimens.

6. Numerical Simulation Setup

Quasi-static numerical analysis of the wrapping origami structures was conducted using Abaqus/Explicit. Due to the geometric mismatch of the wrapping origami structures after offset creases, it cannot be modeled directly. Therefore, the simulation involves two sequential steps, as depicted in Figure S6A. In the first step, the flattened model with offset creases was loaded to the initial pre-folding angle. Crease wide δ was enlarged to 0.75 mm to avoid making crease mesh grids too small and the radius of the hole was adjusted proportionally. Due to the deformation of the crease being mainly plastic, a crease slightly wider than the required width will not have a great effect on the mechanical properties of the crease, which can be verified by comparing the experimental and simulation results later. During preloading, the pressure was applied at the creases to form the valley and mountain line, with the loading by the rigid molds without crease offsets above and below the model. For the second step, the final geometry from the first step was imported. One side of the loading frame was kept fixed, while the other side was loaded. Material properties are assigned based on the results in Table S3 and Figure S3, where stainless steel was treated as elastic and PET as elastic-plastic. A quadrilateral shell

element with reduced integration, S4R, was utilized in all panels using a mesh size of 1.2 mm through convergence tests. Self-contact was also defined for the wrapping structures. Similar to the experimental procedure, the reaction force results from the simulation removed the value caused by the kinetic energy of the boundary frames. The simulation results of the structures with square hubs are shown in Figures S6B-D and compared with the experimental ones, which show excellent correspondence and exhibit multi-stable behaviors.

The flattened pattern of the four-layer hexagonal and octagonal wrapping origami structures holds consistent circumscribed circle diameters d_m with the square hub of 342.1 mm (a=30 mm), yielding varying compression displacement. Table S2 shows the displacement modification results by the creases offset method. The simulation modeling and loading of the hexagonal and octagonal wrapping patterns follow the same approach are shown in Figures S6E,F. It is noteworthy that while one end of the frame is fixed, the other ends lying in the xOy-plane are limited to slide in the plane only to ensure the folding of the frame with a single degree of freedom.

7. Deformation Mechanisms under Different Stiffness Ratios

The deformation result of the smallest stiffness ratio (R_{k1} =10.5) is shown in Figure S7A. The deformation process could be equivalent to the loading of point O as shown in Figure S7B. The von Mises stress contour maps illustrated that panel bending is the primary feature of deformation, concentrated in the trapezoidal area where two traveling hinges formed, grew, and ultimately disappeared in the tightly wrapped state, as shown in Figure S7C. Furthermore, the deformation mechanism of the trapezoidal panels was established, where the parallel traveling creases of PM and PN generated from point B_1, gradually tilted into PA_1 and PQ and finally converged to the A_1Q line, which is illustrated in Figure S7C.

The deformation results corresponding to the experimental specimen under the largest stiffness ratio parameter (R_{k2} =1801.0) is displayed in Figure S8A. Different from the folding process under the small stiffness ratio, the deformation of the wrapping structure is primarily composed of crease bending in the rigidly folding stage, as well as crease bending and torsion deformation mode in the non-rigidly folding stage, as revealed by the local crease deformation. The independent creases and panels were numbered as shown in Figure S8B. The strain energy of each crease and panel by numerical simulation was illustrated in Figure S8C, indicating that the energy of creases is higher than that of panels and that the energy of creases C4 and C7 are

higher than others. Therefore, it can be concluded that under the largest stiffness ratio parameters, the geometric incompatibility of the structure is primarily adapted to the torsional deformation of creases C4 and C7. Subsequently, an equivalent deformation mechanism model was established, which proved that torsion deformation is necessary. Since the panels P1, P2, and P3 and the connected creases are not significantly deformed after state I, it can be assumed that these panels remain fixed while only panels P4 and P5 rotate, as illustrated in Figure S8D. The folding between two stable states split an angle at crease C7 whose value first increased and then decreased with the folding process. Additionally, crease C3 is also slightly twisted in the non-rigid stage, and panel P3 is allowed to rotate while creases C3, C4, and C7 are twisted simultaneously as shown in Figure S8E. Both the deformation mechanism of a single crease torsion and multiple creases torsion explains the necessity for the twist of the wide creases in non-rigid ranges under a large stiffness ratio.

8. Kinematic Analysis of the Hexagonal and Octagonal Wrapping Origami

The crease patterns of the four-layer hexagonal and octagonal structures are shown in Figures 4A,B, respectively. For these four-layer wrapping origamis, the Truss method can be used to analyze the kinematic behavior with some extra work. Taking the four-layer hexagonal structure as an instance, a half-freedom point G has been added to the centerline of the regular hexagon connecting with points A_1, A_3, and A_5, and point G can only move along this centerline(z-axis), as shown in Figure S9. The calculation processes of the four-layer hexagonal and octagonal structure are respectively shown in Figures S9, S10. To confirm that the four-layer hexagonal and octagonal wrapping origami structures can reproduce the folding behaviors of the corresponding two-layer and three-layer ones, same analysis applies to the two-layer and three-layer ones. The kinematic curves of the two-, three-, and four-layer hexagonal and octagonal wrapping origmai can be obtained. Figures S11A,E show the central layer of the hexagonal and octagonal wrapping origami with dihedral angles, respectively. Similar to the analysis process of the comparisons of the two-, three-, and four-layer square wrapping origami structures, the comparisons of the kinematic properties of the same layer in the two-, three-, and four-layer hexagonal wrapping origami are shown in Figures S11B-D, and the comparisons of the octagonal one are shown in Figures S11F-H. These results show that the kinematic curves of the same layers of the two-, three-, and four-layer hexagonal and octagonal wrapping origami are identical. Thus, the four-layer hexagonal and octagonal wrapping origami can reproduce the kinematic properties of two-layer and three-layer ones.

9. Tessellation of the wrapping origami

The multi-stable extensible wrapping origami strucutre can be tessellated to form larger ones. Taking the two-layer square wrapping origami as an example, its 2×2 tessellated crease pattern is shown Figure S13A. The rotational symmetry of the tessellation is maintained, in other words, the adjacent units are obtained by rotating them 90°. Besides, Figure S13B shows the folding process of a corresonding physcial specimen.

10. Figures S1 to S13

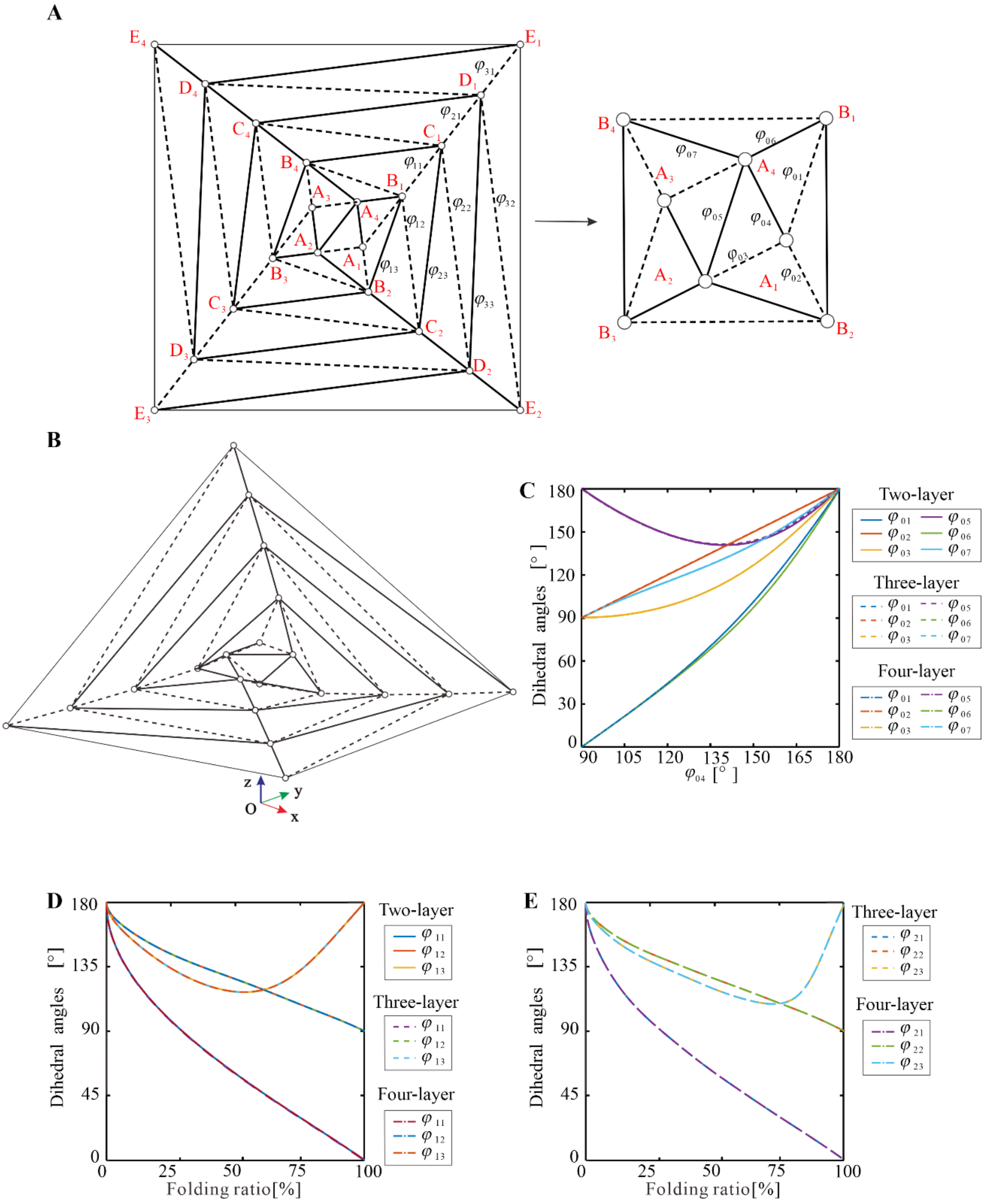

Figure S1. The kinematic analysis of the square wrapping origami. A) The truss form of the four-layer square wrapping origami in the fully unfolded state. B) The truss form of the four-layer square wrapping origami at the general unfolded state. C) Comparison of the relations between dihedral angles and the input dihedral angle φ_{04}, which is located in the central layer of the two-layer, three-layer, and four-layer square wrapping origami, respectively. D) Comparison of the relations between dihedral angles φ_{11}, φ_{12}, φ_{13}, which are located in layer 1

of the two-layer, three-layer, and four-layer square wrapping origami, and the folding ratio of the two-layer one. E) Comparison of the relations between dihedral angles φ_{21}, φ_{22}, φ_{23}, which are located in layer 2 of the three-layer, and four-layer square wrapping origami, and the folding ratio of the three-layer one.

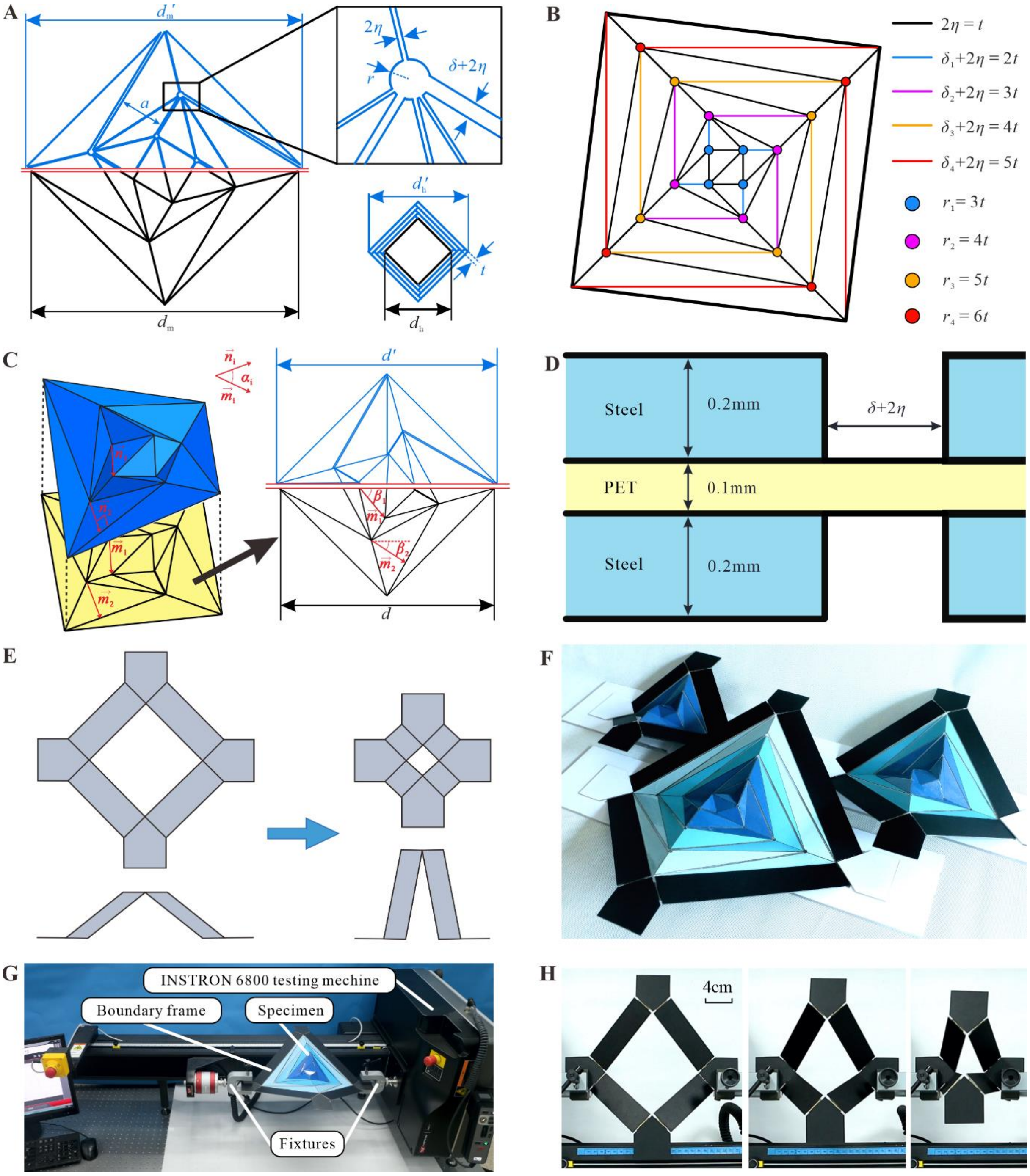

Figure S2. Design and manufacture of physical specimens. A) Geometric parameters of two-layer square wrapping patterns under flattened and tightly wrapping states (black part: theoretical model with zero-thickness; blue part: physical specimen with thickness offset). B) The crease width and circular hole radius of the four-layer square wrapping pattern. C) Displacement modification at the arbitrary folding state. D) The stacked panels with different materials. E) Boundary frame of two-layer pattern in the initial state and tightly wrapped state. F) The specimens with frames of the two-layer, three-layer, and four-layer wrapping origami

patterns at their initial states. G) The setup of the folding experiments. H) The folding process of the boundary frame only.

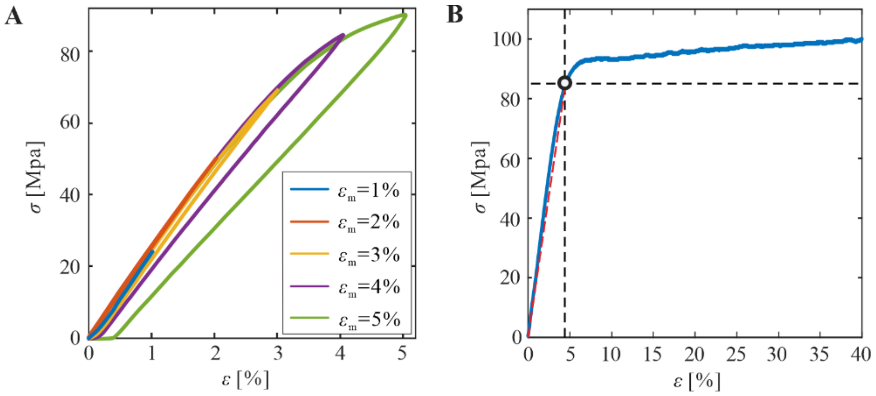

Figure S3. Material properties of PET. A) The residual strain of PET after unloading from different strain ε_m. B) The complete material tensile curve of PET. The black dotted lines indicate the location of the yield stress and the corresponding strain. The red dotted line represents the phase of linear elasticity.

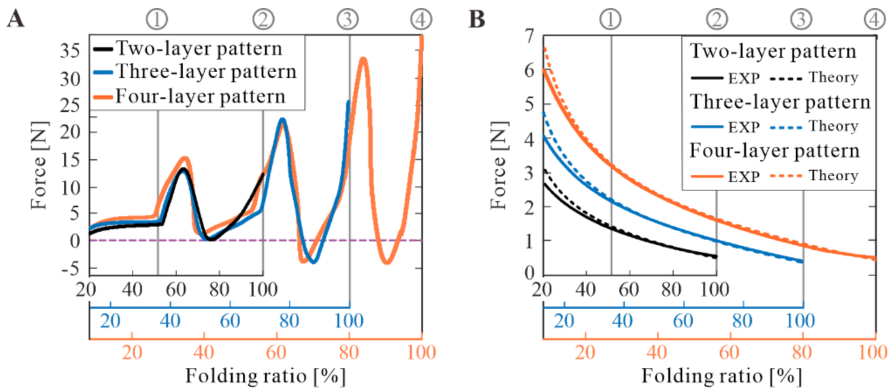

Figure S4. Modification of eliminating gravity effects. A) The experimental force versus folding ratio curves before eliminating specimens' gravity of two-layer, three-layer, and four-layer square wrapping patterns. B) Theoretical modification and experimental loading results of boundary frames corresponding to three specimens.

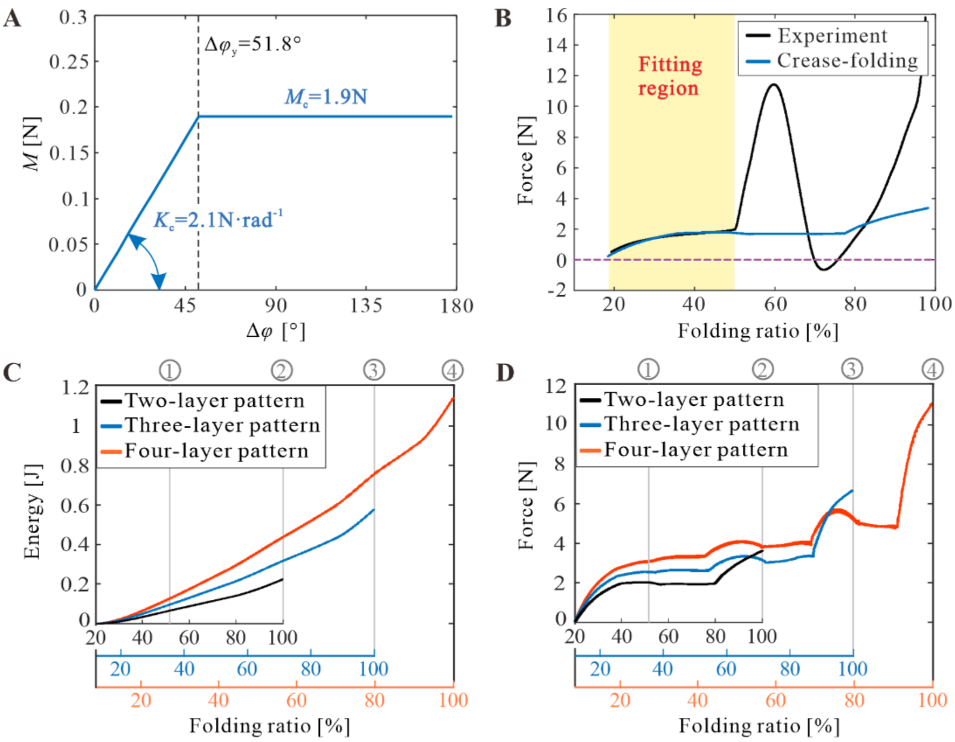

Figure S5. The energy and force caused by crease folding. A) The relationship of the elastic perfectly-plastic moment versus rotation of the creases. B) The fitting of creases moment based on the two-layer square wrapping structure. C) Theoretical folding energy of the two-layer, three-layer, and four-layer wrapping origami patterns. D) The theoretical folding force of the three patterns.

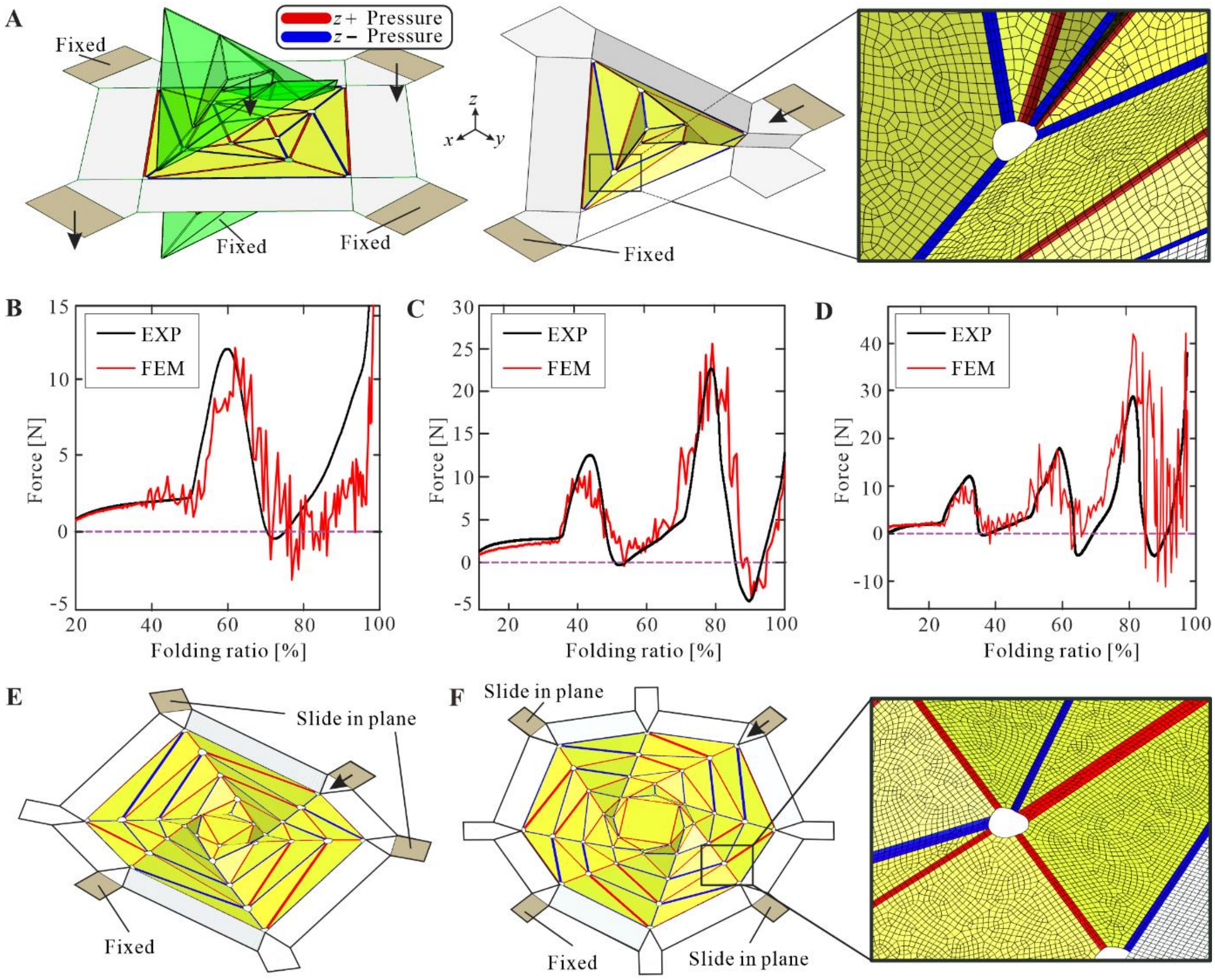

Figure S6. Numerical simulation setup and results. A) Numerical simulation modeling and setup of the two-layer square wrapping pattern. B-D) Experimental and simulation force versus folding ratio curves of two-layer, three-layer, and four-layer square wrapping patterns. E,F) Numerical simulation modeling and setup of the four-layer hexagonal and octagonal wrapping patterns.

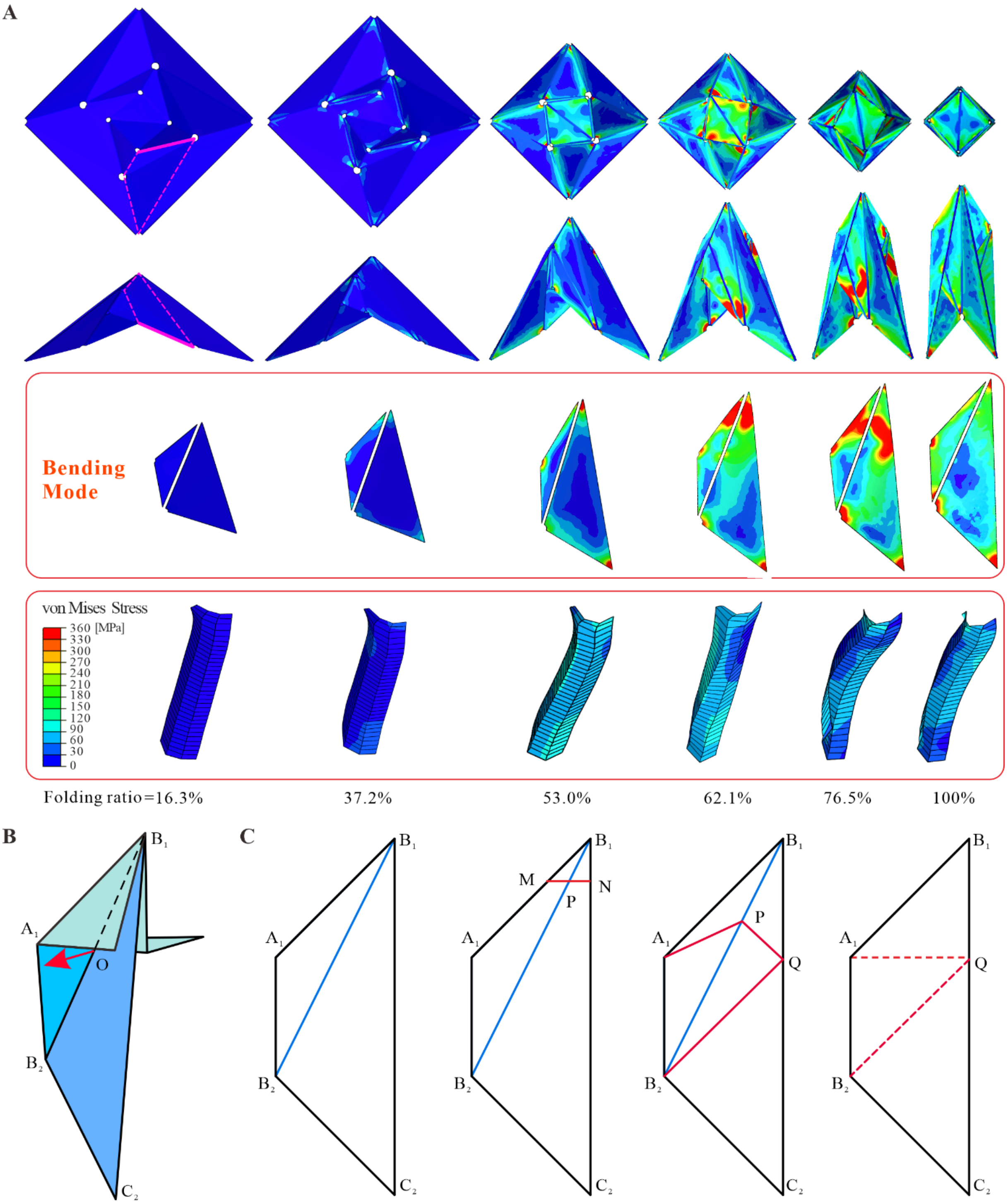

Figure S7. Deformation mechanism under the smallest stiffness ratio (R_{k1} =10.5) of the two-layer origami. A) The folding process and local feature deformation under bending mode. B) Equivalent loading in the trapezoidal area. C) Modeling of travelling hinges, from B_1 to PM-PN to PA_1-PQ to A_1Q.

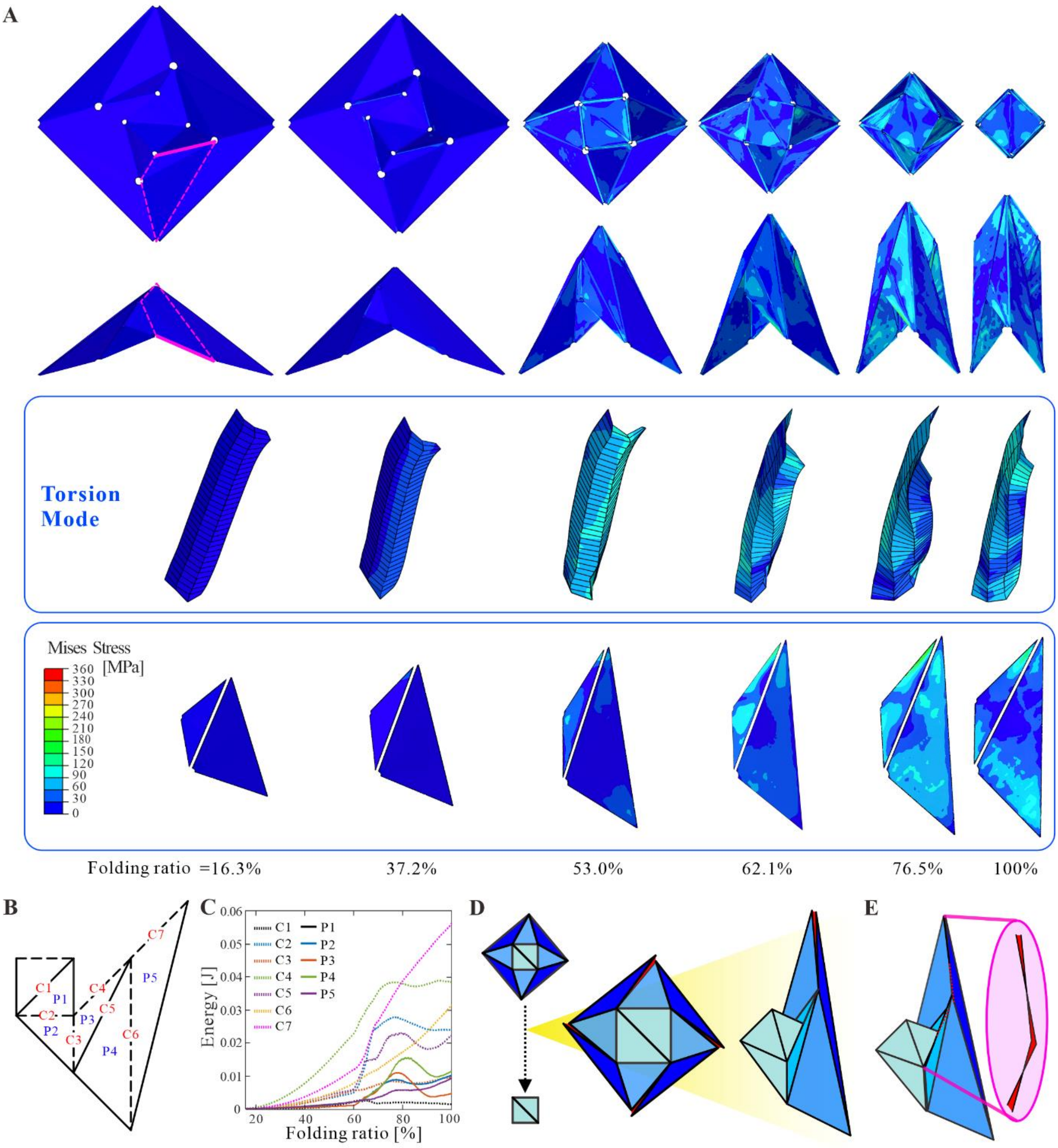

Figure S8. Deformation mechanism under the largest stiffness ratio (R_{k2} =1801.0) of the two-layer origami. A) The folding process and local feature deformation under torsion mode. B) Creases and panels number of the quarter section. C) Energy versus folding ratio curve of creases and panels. D) Deformation mechanism of a single crease torsion (C7). E) Deformation mechanism of multiple creases torsion (C3, C4, and C7).

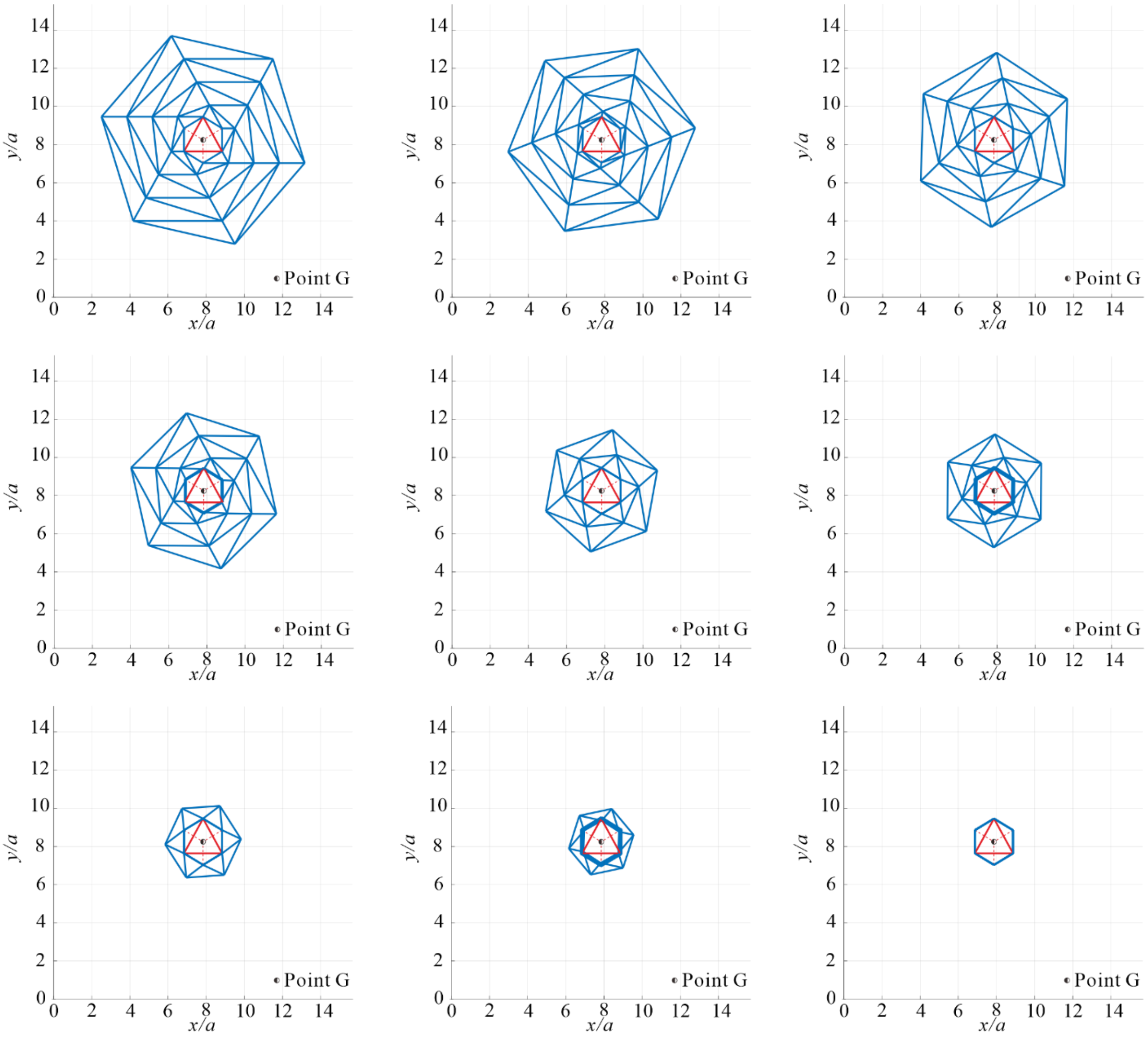

Figure S9. The calculation process of the four-layer hexagonal wrapping origami based on the Truss method.

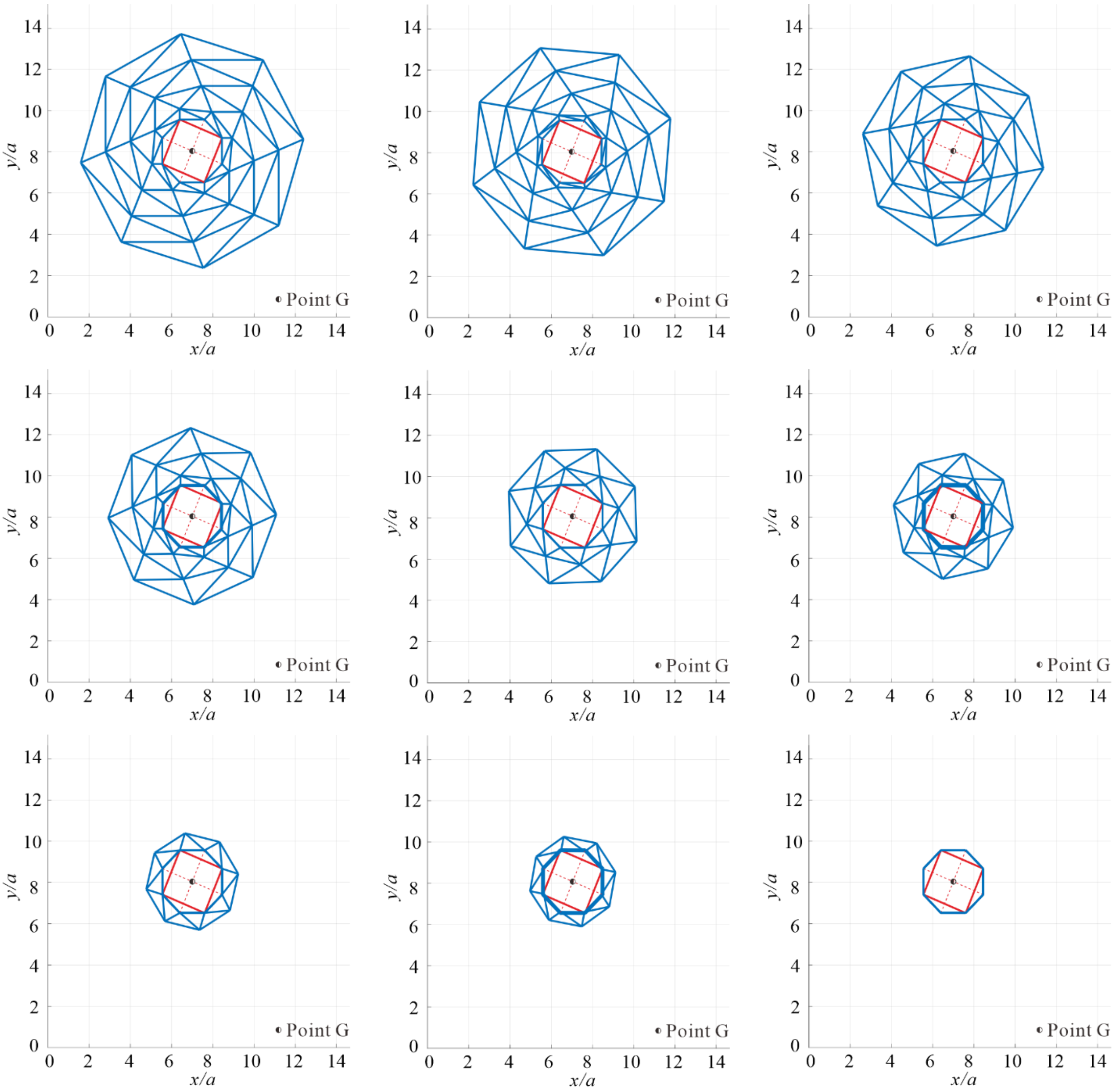

Figure S10. The calculation process of the four-layer octagonal wrapping origami based on the Truss method.

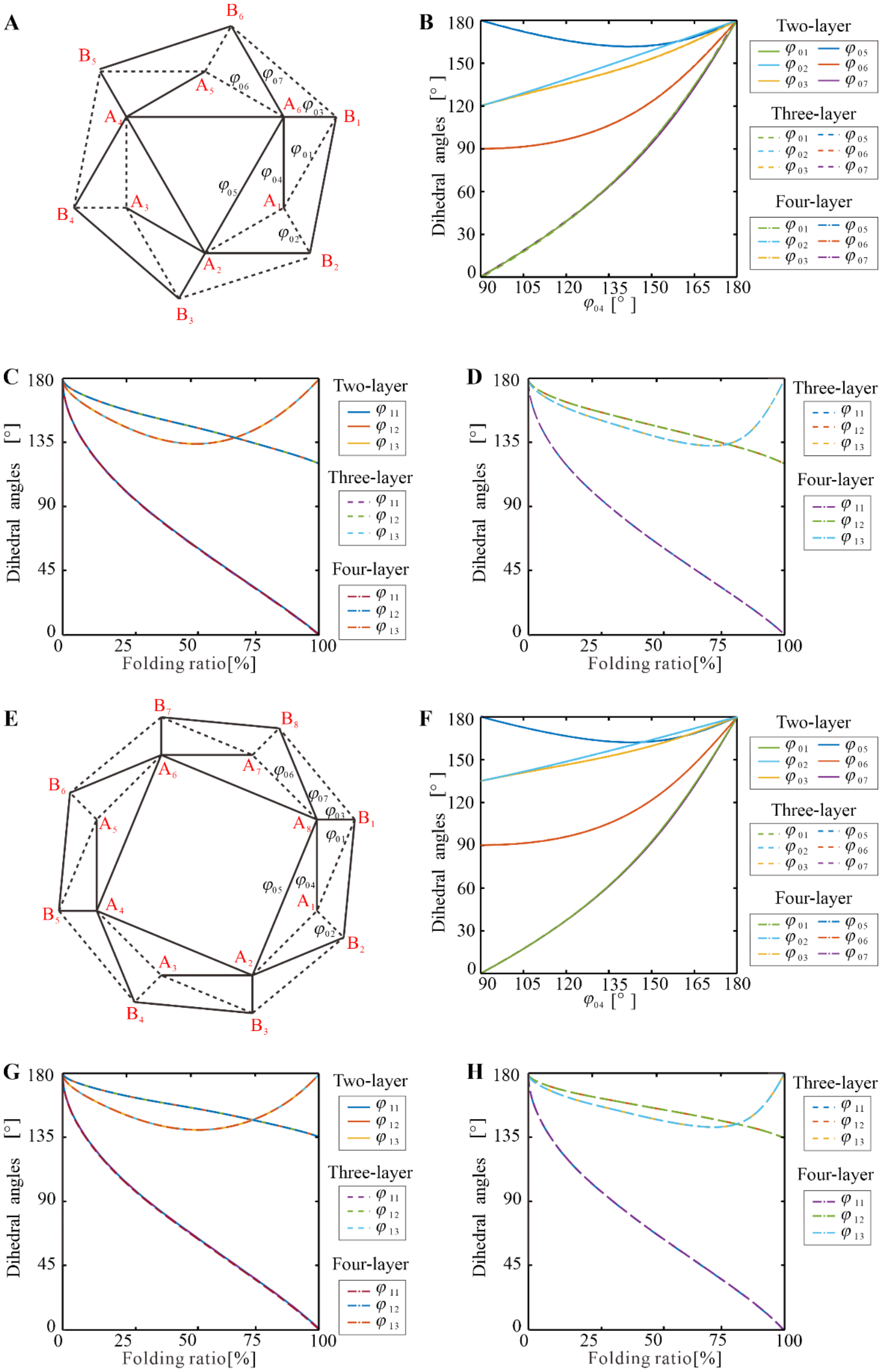

Figure S11. Comparison of kinematic curves between different layers. A) The central layer of the hexagonal wrapping origami. B) Comparison of the relations between dihedral angles and the input dihedral angle φ_{04}, which is located in the central layer of the two-layer, three-layer, and four-layer hexagonal wrapping origami, respectively. C) Comparison of the relations

between dihedral angles φ_{11}, φ_{12}, φ_{13}, which are located in layer 1 of the two-layer, three-layer, and four-layer hexagonal wrapping origami, and the folding ratio of the two-layer one. D) Comparison of the relations between dihedral angles φ_{21}, φ_{22}, φ_{23}, which are located in layer 2 of the three-layer, and four-layer hexagonal wrapping origami, and the folding ratio of the three-layer one. E) The central layer of the octagonal wrapping origami. F) Comparison of the relations between dihedral angles and the input dihedral angle φ_{04}, which is located in the central layer of the two-layer, three-layer, and four-layer octagonal wrapping origami, respectively. G) Comparison of the relations between dihedral angles φ_{11}, φ_{12}, φ_{13}, which are located in layer 1 of the two-layer, three-layer, and four-layer octagonal wrapping origami, and the folding ratio of the two-layer one. H) Comparison of the relations between dihedral angles φ_{21}, φ_{22}, φ_{23}, which are located in layer 2 of the three-layer, and four-layer octagonal wrapping origami, and the folding ratio of the three-layer one.

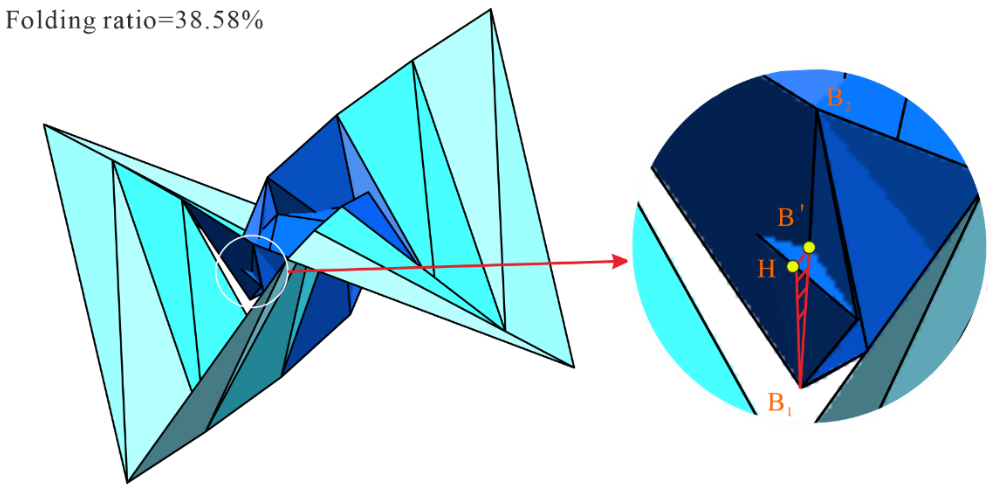

Figure S12. The partially enlarged view of panel penetration of the four-layer hexagonal wrapping origami.

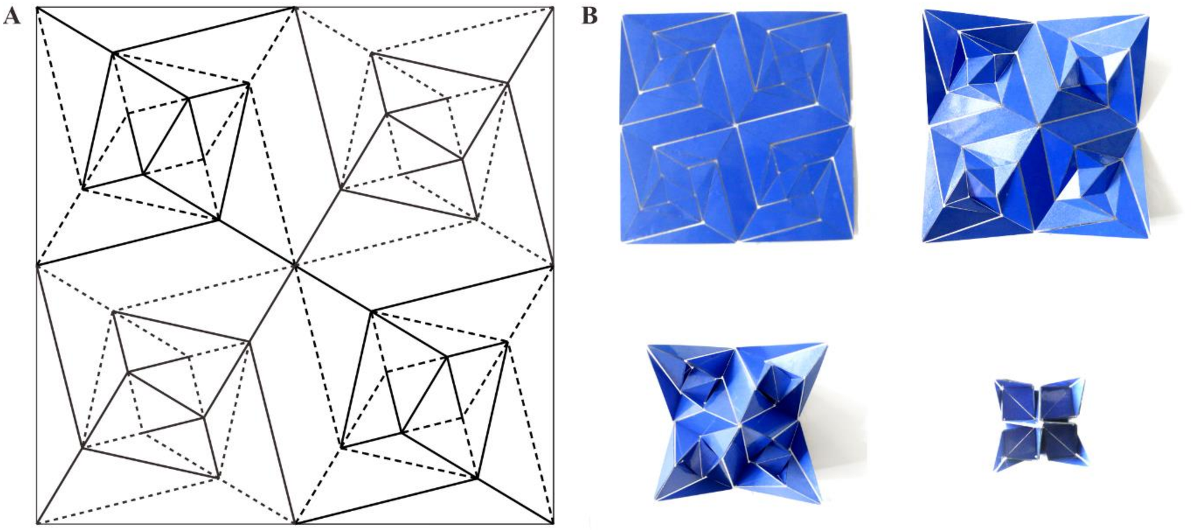

Figure S13. The 2×2 tessellated wrapping origami. A) Crease pattern. B) Folding process of the physical specimen.

11. Tables S1 to S5

Table S1. Coordinates of vertices [mm].

Vertex	*x*	*y*	*z*
A_1	-19.65885647	-11.51831188	94.12890249
A_2	-19.65885792	18.48168554	94.12890339
A_3	10.34114229	18.48168874	94.12890209
A_4	7.22141047	-8.39857823	81.17828303
B_1	-45.28107689	-11.51831446	78.52435613
B_2	-19.65884981	35.70504389	118.69220587
B_3	26.56260093	27.88245357	70.70903619
B_4	16.03400088	-34.4345272	93.19775105
C_1	-69.56707181	-44.43386597	67.26572549
C_2	-51.54888111	54.21779818	139.67562678
C_3	46.88578548	64.76081765	65.5172749
C_4	51.74086551	-56.76411088	88.05874873
D_1	-93.56830937	-78.03254241	57.51536461
D_2	-84.08076297	73.18234697	159.2210365
D_3	67.67595564	101.5705578	61.93790971
D_4	87.35507202	-78.81099436	81.30871305
E_1	-117.41527151	-111.97321896	48.60963132
E_2	-116.93707656	92.39878448	177.96004396
E_3	88.72808455	138.30606779	59.24173639
E_4	122.88567579	-100.70172184	73.67119123

Table S2. The distance between the two diagonal vertices at different states of kinematic models and physical specimens [mm].

		d_m	d_m'	d_0	d_0'	d_h	d_h'	d_0'-d_h'
Square hub	Two-layer	174.9	178.3	153.1	156.8	42.4	46.6	109.9
	Three-layer	258.1	265.7	234.0	242.1	42.4	48.1	193.2
	Four-layer	342.1	356.1	316.7	330.2	42.4	49.5	280.5
Hexagonal hub	Two-layer	182.2	185.3	163.7	166.7	72.4	75.8	90.9
	Three-layer	261.0	267.6	241.0	247.6	72.4	77.0	170.6
	Four-layer	342.1	353.3	321.4	332.5	72.4	78.2	254.3
Octagonal hub	Two-layer	191.2	194.0	175.7	178.3	98.8	102.1	76.2
	Three-layer	264.7	270.8	247.5	253.4	98.8	103.2	150.2
	Four-layer	342.1	352.4	324.0	334.2	98.8	104.2	230.0

Table S3. Material properties of PET and 304 steel.

	ρ [g cm^{-3}]	E [GPa]	σ_s [MPa]
PET	1.37	1.97	83.9
304 steel	7.93	183	205

Table S4. The masses of the specimens and the frames [g].

	**Specimen**	**Boundary frame**
Two-layer pattern	54	538
Three-layer pattern	114	741
Four-layer pattern	205	977

Table S5. Stiffness ratios under different panel thicknesses.

Steel thickness [mm]	0.01	0.02	0.03	0.04	0.05	0.1	0.15	0.2
K_p [N rad^{-1}]	22	54	96	148	214	794	1922	3782
Stiffness ratio R_k	10.5	25.7	45.7	70.5	101.9	378.1	915.2	1801.0

12. Legends for Movies S1 to S3

Movie S1.

Folding process of the four-layer square, hexagonal, and octagonal wrapping origami in Figures 1C,E,G.

Movie S2.

Folding simulation of the four-layer square wrapping origami in Figure 2D.

Movie S3.

Loading experiments of the two-layer, three-layer, and four-layer square wrapping origami in Figures 3A-C.

13. References

[1] S. Wang, Y. Gao, H. Huang, B. Li, H. Guo, R. Liu, *Mech. Mach. Theory*. **2022**, 167, 104512.

[2] J. S. Ku, E. D. Demaine, *J. Mech. Robot.* **2016**, 8, 031003.

[3] S. A. Zirbel, R. J. Lang, M. W. Thomson, D. A. Sigel, P. E. Walkemeyer, B. P. Trease, S.P. Magleby, L. L. Howell, *J. Mech. Design.* **2013**, 135, 111005.
